# Assessing the effects of population-level political, economic and social exposures, interventions and policies on inclusive economy outcomes for health equity in high-income countries: a systematic review of reviews

**DOI:** 10.1186/s13643-023-02429-5

**Published:** 2024-02-08

**Authors:** Anna K. Macintyre, Deborah Shipton, Shifa Sarica, Graeme Scobie, Neil Craig, Gerry McCartney

**Affiliations:** 1 Place and Wellbeing, Public Health Scotland, Edinburgh, EH12 9EB UK; 2 School of Social & Political Sciences, University of Glasgow, Glasgow, UK

**Keywords:** Health equity, Inclusive growth, Inclusive economy, Economic policy, Systematic review of reviews, Umbrella review, High-income countries

## Abstract

**Background:**

A fairer economy is increasingly recognised as crucial for tackling widening social, economic and health inequalities within society. However, which actions have been evaluated for their impact on inclusive economy outcomes is yet unknown.

**Objective:**

Identify the effects of political, economic and social exposures, interventions and policies on inclusive economy (IE) outcomes in high-income countries, by systematically reviewing the review-level evidence.

**Methods:**

We conducted a review of reviews; searching databases (May 2020) EconLit, Web of Science, Sociological Abstracts, ASSIA, International Bibliography of the Social Sciences, Public Health Database, Embase and MEDLINE; and registries PROSPERO, Campbell Collaboration and EPPI Centre (February 2021) and grey literature (August/September 2020). We aimed to identify reviews which examined social, political and/or economic exposures, interventions and policies in relation to two IE outcome domains: *(i) equitable distribution of the benefits of the economy* and *(ii) equitable access to the resources needed to participate in the economy*. Reviews had to include primary studies which compared IE outcomes within or between groups. Quality was assessed using a modified version of AMSTAR-2 and data synthesised informed by SWiM principles.

**Results:**

We identified 19 reviews for inclusion, most of which were low quality, as was the underlying primary evidence. Most reviews (*n* = 14) had outcomes relating to the *benefits of the economy* (rather than *access to resources*) and examined a limited set of interventions, primarily active labour market programmes and social security. There was limited high-quality review evidence to draw upon to identify effects on IE outcomes. Most reviews focused on disadvantaged groups and did not consider equity impacts.

**Conclusions:**

Review-level evidence is sparse and focuses on ‘corrective’ approaches. Future reviews should examine a diverse set of ‘upstream’ actions intended to be inclusive ‘by design’ and consider a wider range of outcomes, with particular attention to socioeconomic inequalities.

**Supplementary Information:**

The online version contains supplementary material available at 10.1186/s13643-023-02429-5.

## Introduction

Despite considerable research and policy attention over the past four decades, substantial inequalities in mortality and morbidity persist [1–3], and relative inequalities have increased across most European countries [4]. In several high-income countries, particularly the UK and the USA, overall life expectancy trends have stalled since 2012 [1, 5–8], with rising mortality in the most deprived areas [9].

Reducing health inequalities demands cross-sectoral activity [10] and requires action to reduce inequalities in the ‘fundamental causes’ of income, wealth and power [11, 12]. The past decade has seen the growth of ‘Health-in-all-Policies’ (HiAP) approaches, which aim to consider the health and health inequalities impacts of policies in education, transport, the environment and so on [13, 14]. Tackling health inequalities requires action on *economic* policy making [15], including changes to social security and taxation systems, reducing poverty, eradicating low pay, increasing employment, improving the quality of work and reducing precarity [1, 2, 8, 15]. Action for health equity necessitates that public health engage with economic policy development [1, 2, 15–22].

### Inequality and its damaging effects on health and the economy

The idea that the current economic paradigm is not delivering for society, or the economy, is increasingly recognised by economists and beyond. Alongside stalling life expectancy, increasing economic inequality is a critical challenge for the twenty-first century [23, 24]. Widening inequalities are negatively associated with health outcomes [1, 2], as well as a range of other indicators of societal progress including education, crime, social capital and trust and social unrest [25, 26]. Further, inequality is damaging to the economy and economic growth itself [26–30].

The past decade has seen significant social and economic change. The austerity policies widely implemented across Europe after the financial crisis of 2008 were associated with significant impacts on health [8, 31–33]. The social and economic impact of the COVID-19 pandemic further highlighted the health impact of economic inequalities [21, 34, 35]. Fundamental questions have been raised about the objectives of the economic system, increased awareness of pre-pandemic inequalities and support for government intervention to address inequalities and promote recovery [23, 35, 36]. There have been calls to ‘build [the economy] back better’ and ‘build back fairer’ in efforts to address economic injustices and health inequalities [21, 23, 37, 38].

### Alternative economic models for health equity

These considerable societal shifts since the mid-2000s have disrupted the status quo, garnered international policy attention on reducing economic inequality [39] and stimulated significant debate regarding alternative ‘heterodox’ economic approaches [15, 40, 41], albeit with less progress in terms of significant policy change. A wide spectrum of (often overlapping) concepts, frameworks and potential policies have been proposed including inclusive growth, community wealth building, strengthening the foundational economy, a universal basic income, universal basic services and frameworks such as *Doughnut Economics* and the wellbeing economy [23, 40, 42–44]. This review is focused on ‘inclusive economy’, which is similar to the term inclusive growth [44, 45] but, as we are using it, is more ‘neutral’ in relation to economic growth [40], more focused on reforming business models and job quality and considers inclusion important in its own right [44, 46, 47].

### An inclusive economy: attributes, outcome domains and evidence base

There are differing interpretations of inclusive economy, and this review draws on Shipton et al. [48] that defines four attributes of an inclusive economy:

- An inclusive economy is deliberately *designed to be more inclusive*, i.e. the policies, laws, regulations, institutions and governance determine how an economy functions and the extent to which it delivers equity.
- There is greater equity in the distribution of the *benefits of an economy*, such as goods and services, employment, wealth, power and economic value.
- There is equitable *access to the resources* needed to participate in the economy including good health, social support and access to education and training.
- The economy functions within the limits of *planetary resources* [48].

This review focuses on two of these attributes (Table 1).

**Table 1:** Inclusive economy outcome domains focused on in this review^a^ (Shipton et al. 2021b) [48]

• The distribution of the ***benefits of the economy***, specifically the following: (a) essential goods and services (e.g. water, electricity, housing or digital connectivity), (b) economic inclusion (e.g. access to stable employment, adequate and stable income), (c) assets that confer economic power (e.g. wealth, capital or social connections) and (d) the value conferred on different parts of the economy (such as female-dominated or unpaid sectors) • ***Access to the resources*** needed to participate in the economy, e.g. access to early years experiences, health, education, training, employment, finance	

^a^For the purposes of this paper, we distinguish between these ‘inclusive economy (IE) outcome domains’ and ‘review-level outcomes’ which are those specific outcomes considered in the included reviews (see the ‘Data synthesis: applying synthesis without meta-analysis (SWiM) to a review of reviews’ section)

The policy rhetoric regarding an inclusive economy has not yet translated into coherent policy action. There is a lack of evidence about what would deliver an inclusive economy [49], and policy-making decisions are not yet underpinned by a systematic understanding of the available evidence.

To address such a gap requires a broad overview of a wide range of evidence. Reviews of reviews (sometimes called ‘umbrella reviews’ [50]) are widely used in medicine and public health to synthesise evidence across a topic area to inform decision-making [51, 52]. Given the breadth of potential policies and interventions related to the concept of an inclusive economy, a systematic review of primary evidence would not provide the required scope, and so a review of reviews was indicated to capture diverse evidence across this topic as well as key gaps.

To the best of our knowledge, a review of the review-level evidence on the effects of exposures, interventions and policies on inclusive economy outcomes does not yet exist. This missing synthesised evidence is a vital step in efforts to advance effective action towards inclusive economies and address health inequalities. Our review of reviews is intended to address this gap.

## Objectives and review question

We aimed to systematically collate and synthesise existing review-level evidence on the effects of political, economic and social exposures, interventions and policies on inclusive economy outcomes to address the following objectives:

- To synthesise review-level evidence on the effects of exposures, interventions and policies on inclusive economy outcomes
- To assess the quality of existing reviews and areas where reviews are missing to inform future work in this area

The review question is as follows: *What are the effects of population-level political, economic and social exposures, interventions and policies on inclusive economy outcomes in high-income countries?*

## Methods

### Study design

We conducted a review of reviews [53]. A pre-registration form was completed on the Open Science Framework in September 2020 (DOI: 10.17605/OSF.IO/SWT4E) and the full protocol published on SocArXiv papers in January 2021 (10.31235/osf.io/dctk5) (Supplementary File 1 details amendments to the protocol).

The protocol was written in accordance with the Preferred Reporting Items for Systematic review and Meta-Analysis Protocols (PRISMA) 2015 [54] and results reported using the PRISMA 2020 statement [55] (Supplementary Files 2a and b).

### Inclusion and exclusion criteria

- Inclusion criteria are as follows (amended from protocol, see Supplementary File 1):
- *Population*: Humans only and high-income countries only
- *Intervention(s)/exposure(s)*: Any social, political and economic intervention, policy or exposure
- *Comparison*: Eligible reviews had to include primary studies which compared IE outcomes either within or between groups.
- *Outcome(s)*: Eligible reviews had to include at least one of the following inclusive economy outcomes:

(i) Equitable distribution of the *benefits of the economy*
(ii) Equitable *access to the resources* needed to participate in the economy (see Table 1 for more details).

- *Study design*: Reviews or systematic reviews of empirical studies of quantitative and/or qualitative nature
- *Publication year, language and status*: No publication year restrictions, English only, peer-reviewed and grey literature

### Exclusion criteria are as follows:

- Book reviews and books
- Scoping reviews or commentaries

### Information sources

We searched the following:

- Eight bibliometric databases from inception to May 2020 (EconLit (ProQuest); Web of Science; Sociological Abstracts (SocAbs, ProQuest); Applied Social Sciences Index and Abstracts (ASSIA, ProQuest); International Bibliography of the Social Sciences (IBBS, ProQuest); Public Health Database (ProQuest); Embase (Ovid); MEDLINE (Ovid))
- Google Scholar and 12 governmental and non-governmental organisational websites (Centre for Local Economic Strategies; Joseph Rowntree Foundation; Scottish Government; UK Government; Wellbeing Alliance; Research Papers in Economics; What Works Scotland; Fraser of Allander Institute; Royal Society of Arts; The Organisation for Economic Cooperation and Development; Institute for Public Policy Research; Health Foundation)
- Systematic review registries and evidence databases (PROSPERO, the Campbell Collaboration and EPPI Centre)

We undertook hand searching and contacted relevant experts.

### Search strategy

Full details of the pilot searches, the search terms and search strategy are available in the protocol. (see Supplementary File 3 for an example search strategy).

### Data management

Search results from bibliometric databases were imported to RefWorks and de-duplicated. Results from grey literature searching were imported into Sciwheel and de-duplicated. References were then imported into Covidence (https://www.covidence.org) for screening and data extraction. Additional de-duplication of studies was also performed in Covidence.

### Study selection and data extraction

Title, abstract and full-text screening were undertaken by two independent reviewers (SS, DS or GS), and differences were resolved through consensus. A third reviewer was involved where necessary.

See Supplementary File 4 for the data extraction fields. Two independent reviewers extracted data for 12 reviews (SS, DS or GS), and 1 reviewer extracted data for the remaining 8 reviews (SS) which were checked by a second reviewer (DS or GS).

For the comparator criteria, it became clear that:

(i) Reviews sometimes included primary studies with a mixture of primary study designs, i.e. some that had a comparator and some which did not.
(ii) Not all reviews reported primary studies’ study designs.

Therefore, further data was extracted on how many of the included primary studies with an IE outcome had some form of comparator. Where this information on study design was not available from the review, this data was extracted from primary studies (title, abstract and/or full text where required) for reviews that included < 50 primary studies with IE outcomes.

### Quality assessment

Quality assessment of reviews used a modified version of A Measurement Tool to Assess Systematic Reviews-2 (AMSTAR-2) [56] (Supplementary File 4). Quality assessment was undertaken by two independent reviewers (SS or DS or GS) in Covidence and disagreements resolved through consensus. AMSTAR-2 is designed to be adapted [56]. As so few of the included reviews undertook meta-analysis, items 11 and 15 were not considered critical weaknesses. Further, given the cross-disciplinary nature of the included reviews, we did not code item 7 *(*‘Did the review authors provide a list of excluded studies and justify the exclusions?’) as critical weaknesses (see Supplementary File 7 for the quality assessment scores where item 7 is, and is not, coded as a critical weakness).

### Data synthesis: applying synthesis without meta-analysis (SWiM) to a review of reviews

Due to the variety of outcomes, meta-analysis was deemed inappropriate, and so we report our findings applying the Synthesis Without Meta-analysis (SWiM) guidelines [57] as far as possible.

Reviews were grouped by ‘review-level outcome’ and mapped to the two ‘IE outcome domains’ (Table 1). Given there was no consistent reporting of effect sizes, vote counting based on effect direction (ED) was chosen as the standardised metric [58]. For each review, the ED was coded at the review-level based on the review authors’ synthesis as follows:

(i) Beneficial impact on IE outcomes
(ii) Harmful impact on IE outcomes
(iii) No change/mixed effects/conflicting findings/insufficient evidence^1^

One reviewer coded all EDs (AM), these were cross-checked by a second reviewer (DS or GS) and differences were resolved by consensus. Where a review included more than one exposure/intervention/policy, we have coded the ED separately for distinct exposures/interventions/policies. Where the denominator of included primary studies with an IE outcome was unclear or where there were no included primary studies with a comparator for a particular exposure/intervention/policy, an ED was not coded. Modified^2^ ED plots have been chosen to visually represent the results (Tables 3 and 4).

### Confidence in cumulative evidence

We were unable to apply GRADE [59] (see Supplementary File 5 for details).

## Results

**Fig. 1:**
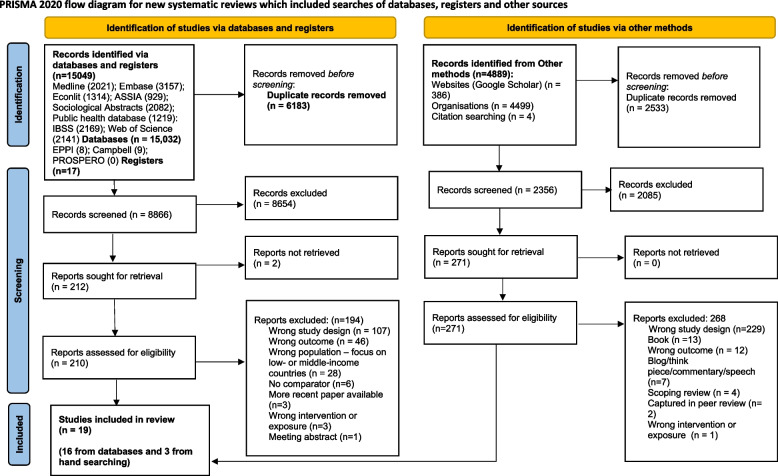
PRISMA 2020 flow diagram [55]

We identified 19 reviews for inclusion^3^ (Fig. 1). See Supplementary Files 6 and 7 for the included reviews and Supplementary File 8 for the excluded reviews. The earliest included review was published in 2005 and the latest in 2020. We excluded duplicate primary studies (only four primary studies occurred in more than one review, Supplementary File 6). Twelve reviews referred to an underlying theory or framework or suggested how the intervention/exposure might impact on outcomes, whilst seven reviews did not refer to theory.

### Summary of included reviews

The majority of the included reviews (*n* = 14) focused on disadvantaged populations, of which half (*n* = 7) focused on either unemployed adults or working age adults with health problems or disabilities, and the rest focused on specific groups such as low-skilled and migrant workers (*n* = 1), disadvantaged African American young men (*n* = 1), disadvantaged students (*n* = 2), low-income mothers (*n* = 1), homeless families (*n* = 1) and refugees (*n* = 1). The remaining reviews (*n* = 5) considered working age adults or young people or focused at a whole country level.

Of the 19 included reviews, three were high quality, three moderate quality, three low quality and the rest (*n* = 10) critically low quality.

Primary study quality was assessed and/or reported (to some degree) by 11 reviews, using methods ranging from formal quality assessment tools to more general comments (Tables 3 and 4). Of those that reported formal quality scores, two reviews identified primary studies as high/medium quality [60, 61], three reviews considered primary studies as low/medium quality [62–64] and one review reported primary studies as a mixture of weak/moderate/strong quality [65]. Of those that did not report formal quality scores or provide an overall rating, three reviews excluded studies which were at high risk of bias [66], had ‘substantive flaws’ [67] or did not meet validity criteria [68], whilst two reviews critically appraised studies to inform interpretation, but no overall comment on quality was provided [69, 70]. Primary study quality was not reported by eight reviews including one ‘empty’ review which had planned to assess quality, but no studies met inclusion criteria [71].

There were four groups of reviews:

- *n* = 7 reviews: All primary studies had a comparator.
- *n* = 8 reviews: Some primary studies had a comparator, and we could identify the proportion.
- *n* = 3 reviews: Some primary studies had a comparator, but we were unable to identify the proportion.
- *n* = 1 review: Was an empty review (i.e. a review where no studies met inclusion criteria), and so assessing the comparator did not apply.

Across the 19 included reviews, we were able to identify 801 primary studies^4^, of which 525 had inclusive economy outcomes^5^, and of these, at least 60% (315 primary studies) had some form of comparator.

Reviews were mapped to two outcome domains: ‘equitable distribution of the benefits of the economy’ and ‘equitable access to resources needed to participate in the economy’ (Table 1) and grouped reviews by review-level outcomes and by exposure/intervention/policy.

The majority of reviews (*n* = 14) had outcomes relating to the *benefits of the economy*, such as employment and earnings. The remaining reviews (*n* = 5) had outcomes relating to *access to resources to participate* in the economy such as access to education or training. For most of the inclusive economy domains, we found none or only a few reviews (Table 2), and these were mostly of low quality.

**Table 2:** Number (n) of reviews mapped by outcome domain and exposure/intervention/policy (*n* = 19 reviews)

Outcome domain →	Equitable distribution of the benefits of the economy (*n* = 14)							Equitable access to resources needed to participate in the economy (*n* = 5)		
	(a) Essential goods and services e.g. water, electricity, housing or digital connectivity	(b) Economic inclusion				(c) Assets that confer economic power e.g. weatlh, capital, social connections	(d) Different parts of the economy are valued e.g. female dominated or unpaid sectors			
Review-level outcome → Exposure/intervention/policy ↓	(*n* = 0)	Increased employment/return to work (*n* = 10)	Reduced poverty or economic/material hardship (*n* = 3)	Increased earnings (*n* = 1)	Reduced income inequality (*n* = 1)	(*n* = 0)	(*n* = 0)	Improved higher education outcomes (*n* = 3)	Entrepreneurial knowledge & skills (*n* = 1)	Access to active labour market programmes (*n* = 1)
Social security	-	2	2	-	-	-	-	-	-	-
Active labour market programmes	-	7	-	1^a^	-	-	-	-	1	-
Active labour market programmes delivery elements										1
Housing interventions	-	1	-	-	-	-	-	-	-	-
Social networks/social support	-	-	1	-	-	-	-	1	-	-
Broad government policies	-	-	-	-	1	-	-	-	-	-
Outreach and financial aid interventions for higher education	-	-	-	-	-	-	-	1	-	-
Enablers for transition to higher education	-	-	-	-	-	-	-	1	-	-

^a^Kluve et al. [60] examined both employment and earnings which is why the total across the outcomes is 15

We now consider key findings for each outcome domain in turn in terms of (i) the types of outcomes and interventions examined by the included reviews and (ii) the effect directions identified in included reviews structured by review-level outcomes and grouped by intervention.

### Equitable distribution of the benefits of the economy

For the first outcome domain, Shipton et al. identify four types of ‘benefits’ of the economy, namely, (a) provision of essential goods and (b) economic inclusion, (c) assets that confer economic power and (d) different parts of the economy are valued (see Table 1).

#### Types of outcomes and interventions examined by the included reviews

Fourteen reviews examined outcomes that could be categorised as a benefit from the economy, all of which focused on measures of economic inclusion, i.e. employment, income and reduced poverty. No reviews examined outcomes related to any other types of economic benefits (i.e. (a), (c) or (d) in Table 2).

Of those reviews which considered economic inclusion outcomes, more than two-thirds (*n* = 10 reviews) focused on increased employment/return to work as the main outcome of interest. Other economic inclusion outcomes were reduced poverty and economic/material hardship (*n* = 3 reviews), and one review considered ways to increase earnings and one review on reducing income inequality. One review examined both employment and earnings [60]. None of the reviews examined other measures of economic inclusion outcomes such as the social benefits of participation in the economy [48], and so measures of economic inclusion were restricted to ‘traditional’ outcomes, primarily gaining paid employment.

For this IE outcome domain (‘equitable distribution of the benefits of the economy’), the most common types of interventions to deliver economic inclusion considered by the included reviews were various types of active labour market programmes (ALMP) (*n* = 7 reviews) (Table 3). These interventions predominantly focused on supply-side interventions such as skills training or employability support and more limited consideration of demand-side interventions such as wage subsidies or job creation programmes. The next most common intervention type (examined by *n* = 4 reviews) explored was forms of social security (specifically unemployment insurance and disability benefits) and how they relate to economic inclusion. The remaining reviews examined a diverse set of exposures and interventions: social support (*n* = 1 review), housing interventions for homeless families (*n* = 1 review) and government policies to tackle income inequality (*n* = 1 review).

**Table 3:** Effect directions for exposures/interventions/policies on ‘More equity in distribution of the benefits of the economy’

*Inclusive economy outcome domain*: More equity in distribution of the benefits of the economy, specifically economic inclusion (*n* = 14 reviews)							
	Effect direction (ED)			Review characteristics			
Review	Exposure/intervention/policy	Effect direction	Comments on ED	Review quality	PS quality (as reported by review authors)	PS with comparator/PS with IE outcome^a^	Context/population
*2a. Review-level outcome:* Increased employment/return to work							
** (i)** *Intervention/policy*: Social security interventions							
[66]	*Unemployment insurance coming to an end* (i.e. state-administered social security for people who are unemployed which has a time limit and known expiration date)	▲	Transition to employment increased in the month/week that benefits came to an end	High	High ROB studies excluded	12/12	National programmes in European countries, the USA, and Canada Unemployed individuals
[68]	(i) *Increased eligibility for disability benefits*, i.e. relaxed eligibility for income replacement benefits provided by the state to those not in the labour market for more than 3 months due to health problems or disabilities	◄►	RA conclude there is ‘insufficient evidence’, and available evidence is mixed	Low	PS not meeting validity criteria excluded. RA note lack of evidence ‘of a high enough quality’ (p. 1112)	15/16	Canada, Norway, Sweden, the UK Working age adults (16–69 years) with health problems or disabilities
	(ii) *Increased generosity of disability benefits*, i.e. increased generosity of income replacement benefits provided by the state to those not in the labour market for more than 3 months due to health problems or disabilities	▼	RA highlight that lack of high-quality evidence to determine extent of effect				
** (ii)** *Intervention/policy*: Active labour market programmes							
[62]	*Workplace disability management programmes* provided by employers within the workplace setting to address sick leave due to physical or mental ill health	◄►	RA unable to conclude regarding effectiveness due to insufficient data	High	Overall quality of the two non-randomised studies reported as ‘low’	13/13	Interventions in North America only Employees on sick leave due to physical injury, illness or mental health disorders
[71]	Interventions intended to increase economic self-sufficiency and wellbeing of refugees. No restrictions on intervention type and could include employment casework, translation and administrative support, mentorship, interview training and therapy or community support	-	Unable to code ED as no eligible studies identified	High	N/A	N/A	Refugees
[60]	(i) *Skills training interventions* which could include providing technical skills, business skills, literacy and numeracy and non-technical skills, e.g. life skills/soft skills	▲	RA report effect is small	Moderate	Although not a full risk-of-bias assessment, quality of PS assessed as 48% high, 42% medium and 9% low quality (of 113 primary studies, 65 of which were in high-income countries)	113/113	Interventions aimed at young people aged 15–35 years globally (58% HIC, 42% LMIC) We report here on results for HIC only (i.e. 65 studies)
	(ii) *Entrepreneurship promotion interventions* including providing entrepreneurial skills, access to credit (Inc. microfinance), start-up grants and technical support, support for micro franchising	◄►	Lack of effect sizes for HIC, i.e. insufficient data				
	(iii) *Subsidised employment* including wage subsidies and public employment programmes intended to reduce employers’ labour costs	◄►	RA state no effect				
	(iv) *Employment services* including support with job search, mentoring, job counselling and placements and technical/financial support	◄►	RA state effect negligible				
[69]^b^	(i) *Individual case management and job search assistance* such as help with job searches and vocational advice	▲	RA conclude that personal advisers and case management can help participants back to work but selection bias problematic	Critically low	Papers critically appraised but scores not reported and used descriptively to inform interpretation of the results	6/31	National programmes focused on working age adults with long-term illness or disability in the UK only
[70]	(i) *Anti-discrimination legislation*, i.e. to outlaw discrimination and require employers to make reasonable adjustments. All studies focused on the UK’s Disability Discrimination Act (1995, 2005)	◄►	RA conclude lack of evidence which has detected an effect at a population level	Critically low	Papers critically assessed but scores not reported. Review authors comment ‘few studies provided robust evaluations’ (p. 434)	12/30	National programmes in Canada, Denmark, Norway, Sweden and the UK For studies of anti-discrimination legislation — all were focused on UK legislation Working age adults (16–65 years) who are chronically ill or disabled
	(ii) *Workplace adjustments* including legal or financial actions to address the accessibility of work to those who are disabled/chronically ill	▲	RA conclude positive impact but low uptake				
	(iii) *Wage subsidies* for employing people with disabilities including creation of jobs or financial incentives to encourage employers to employ people with disabilities or chronic illness	◄►	RA conclude wage subsidies can be effective if sufficiently generous but can have unintended effects and both positive and negative effects				
	(iv) *Return-to-work (RTW) planning* includes measures to require employers to engage in order to speed up return to work	▲	RA conclude RTW planning can reduce sick leave but uptake by employers low				
[63]^c^	(i) *Cognitive behaviour therapy* delivered by Jobcentre Plus work psychologists over 8 weeks for those anxiety/depression	◄►	RA conclude insufficient evidence	Critically low	One PS assessed as low quality, one as medium quality	2/3	Interventions in the UK to support people receiving ‘incapacity benefit’ to return to work
	(ii) *Work-focused interviews/employability support*, specifically the Pathways to Work programme which involved mandatory work-focused interviews and voluntary engagement in some/all of the following: training, a return-to-work credit, mentoring, job coach, occupational health support, financial advice and a discretionary fund for purchases to support return to work	▲	Effects stronger for women and for those without mental illness				
[67]^d^	(ii) *Vocational advice and support services* intended to provide support with identifying opportunities and obtaining work	◄►	RA state that PS found positive impact, but only study with a control did not identify an effect	Critically low	Quality criteria not clear but PS with ‘substantive flaws’ excluded (p. 1908). RA report quality of observational studies specifically as ‘variable’ (p. 1916)	2/16	Programmes in the UK Individuals of working age (16–59/64 years) with a moderate physical or mental illness/disability
	(iii) *In-work benefits* intended to offer financial incentives to employers to employ those who with a disability or chronic illness	◄►	RA found three (of four) studies found positive impact but uptake low				
** (iii)** *Intervention/policy*: Housing interventions							
[64]	*Housing interventions for homeless families* such as Housing First, rapid, emergency or transitional housing, permanent supportive housing, etc.	▲	RA conclude positive effect but note that mothers were generally not earning a living wage or in stable employment	Moderate	PS quality reported as weak or moderate	6/6	Focused on homeless families in the USA only
*2b. Review-level outcome*: Reduced poverty or economic/material hardship							
** (i)** *Intervention/policy*: Social security interventions							
[65]	*Unemployment insurance*, i.e. state-administered benefit for those who are unemployed	▲	Unable to code EDs for specific interventions	Low	Of the three PS focused on poverty, one assessed as weak, one moderate, one strong	3/3 (12 total PS)	For the three PS focused on poverty *—* North America and Nordic European countries Working population
[73]	(i) *Unemployment insurance* (i.e. state benefit for those who are unemployed) *— generosity of eligibility criteria*, i.e. who it can be received by	▲		Critically low	Not reported	3/15 (33 total PS)	Working age adults OECD countries. For the studies focused on poverty *—* eastern European countries (specifically in the 1990s) and North America
	(ii) *Unemployment insurance* (i.e. state benefit for those who are unemployed) *— level of benefit*, i.e. the extent to which benefits replace wages	◄►	When benefit levels too low poverty not reduced *—* no change				
	(iii) *Unemployment insurance — flexibility eligibility criteria*, e.g. accommodating the working patterns of seasonal or part-time workers	▲	Flexible eligibility criteria associated with preventing material hardship				
** (ii) ***Exposure:* *Social support*							
[61]	*Social support*, specifically *perceived* informal support, either instrumental support (e.g. emergency access to a place to stay, child care, loan of money) or emotional support (e.g. someone to talk to).	▲	Effect does not extend to employment, job quality or wages	Low	Of the studies focused on economic outcomes, all assessed as within the high-quality range	9/9 (65 total PS)	Focused on low-income mothers in the USA only
*2c. Review-level outcome*: Increased earnings							
(i) *Intervention/policy*: Active labour market programmes							
[60]	(i) *Skills training interventions* which could include providing technical skills, business skills, literacy and numeracy and non-technical skills, e.g. life skills/soft skills	◄►	Effect size small and confidence interval marginal suggests no change	Moderate	Although not a full risk-of-bias assessment, quality of PS assessed as 48% high, 42% medium and 9% low quality (of 113 primary studies, 65 of which were in high-income countries)	113/113	Interventions aimed at young people aged 15–35 years globally (58% HIC, 42% LMIC) We report here on results for HIC only (i.e. 65 studies)
	(ii) *Entrepreneurship promotion interventions* including providing entrepreneurial skills, access to credit (inc. microfinance), start-up grants and technical support, support for micro franchising	◄►	RA state lack of effect sizes in HIC meant it could not be analysed, i.e. insufficient data				
	(iv) *Employment services* including support with job search, mentoring, job counselling and placements and technical/financial support	◄►	Effect size small and confidence interval marginal suggests no change				
	(iii) *Subsidised employment* including wage subsidies and public employment programmes intended to reduce employers’ labour costs	▼	Negative effect but small				
*2d. Review-level outcome*: Reduced income inequality							
** (i)** *Intervention/policy*: Government policies to tackle income inequality							
[74]	Government policies to tackle income inequality e.g. fiscal policies, education policies, trade liberalisation and labour market reform	-	Unable to code ED due to lack of data on PS denominator	Critically low	Not reported	Unclear	Unclear. Global in focus but context not specified

Key: Effect direction (ED): upward arrow ▲ beneficial impact on IE outcomes, downward arrow ▼ harmful impact on IE outcomes, sideways arrows ◄► no change/mixed effects/conflicting findings/insufficient evidence. *ED* effect direction, *HIC* high-income country, *IE* inclusive economy, *LMIC* low- and middle- income country, *PS* primary study, *RA* review authors, *ROB* risk of bias

^a^Where the number of PS with an IE outcome is not the same as the total number of PS (i.e. non-IE outcomes were considered by some PS), this is noted in brackets

^b^Clayton et al. [69] also examined three other intervention types: financial incentives, education and training and health condition/impairment management, but each of these did not have any primary studies with a comparator so an ED has not been coded

^c^Hayday et al. [63] also examined rehabilitation programmes, but this did not include any primary studies with a comparator so an ED has not been coded

^d^Bambra et al. [67] also examined three other intervention types: education, training and work placement initiatives, employer incentive initiatives and accessibility interventions, but each of these did not include any primary studies with comparators so EDs have not been coded

#### Effect directions identified in included reviews

We now consider effect directions (EDs) identified in included reviews in the first outcome domain, paying attention to higher quality reviews (Table 3).

##### Review-level outcome: increasing employment/return to work

Most of the included reviews in the economic inclusion outcome area (*n* = 10) examined interventions aimed at increasing employment/ return to work outcomes (Table 3a). There were three different categories of intervention: (i) social security interventions; (ii) active labour market programmes and (iii) housing interventions.

In relation to the effects of *(i) social security interventions* on increasing employment, two reviews looked at slightly different aspects of social security in relationship to movement into employment. One high-quality review found that there was an increase in the exit rate from unemployment (i.e. increase in employment) in the months before the benefit came to an end [66]. However, this review highlighted that there was a lack of evidence to assess whether people accept jobs that they then quickly leave, i.e. the ‘exit rate from re-employment’. The second, a low-quality review, found increased generosity of benefits decreased the likelihood of moving into employment. The same study also looked at the relationship between relaxing eligibility and movement into employment, but there was not enough evidence to draw a conclusion [68].

Seven reviews examined various types of *(ii) active labour market programmes* on increasing employment/return to work. The reviewed programmes were either focused on individuals (such as in-work benefits, skills training (e.g. training in technical/vocational skills, literacy, numeracy, soft skills, employment support services, cognitive behaviour therapy, entrepreneurship promotion programmes) or were aimed at employers (including employer incentives, job creation programmes, anti-discrimination legislation, workplace disability management programmes and workplace adjustments). This variety in intervention type made synthesising across reviews challenging, and so we have considered the effects of active labour market programmes in general.

The two high-quality reviews identified a lack of available evidence. Focusing on workplace disability management programmes, one review found a lack of studies which provided effect sizes and high risk of bias in the two non-randomised studies [62], whilst another, with a focus on ALMP interventions (including ALMP-type programmes) which might improve economic outcomes for refugees, found no primary studies that met their inclusion criteria [71]. One moderate quality review found skills training could have positive (though small) effects on employment outcomes for young people, whilst there were less conclusive effects for three other interventions (entrepreneurship promotion interventions, subsidised employment and employment services) [60].

The remaining four reviews (all critically low quality) identified a variety of effects which appeared to depend on programme type. The low quality of the reviews, and the general lack of quality scores for primary studies, necessitates cautious interpretation of effect directions. Personal advisors and case management [69] as well as workplace adjustments and return-to-work planning could have positive effects [70], but impact was limited for both by low uptake. There was also some limited evidence that work-focused interviews, and employability support could promote employment outcomes [63]. For other programme types, there was a lack of evidence of effect, including for anti-discrimination legislation [70] and cognitive behaviour therapy [63]. There were mixed findings for the other interventions: wage subsidies could be effective but needed to be sufficiently generous and could have unintended consequences (such as excluding people with disabilities from the wider labour market) [70], vocational advice and support services showed positive effects in some studies but not when limited to only the studies with controls [67] and in-work benefits were found to be effective by several studies, but impact was limited by low uptake [67].

Finally, one moderate quality review was the only one to consider the impact of *(iii) housing interventions* on employment outcomes and found some beneficial impact on employment status of housing interventions for homeless families in the USA [64]. However, participants were often not earning a wage they could live on and continued to experience employment instability. Although this review focuses on a particularly marginalised group and draws on a small set of mixed quality primary studies, it is important in indicating the potential for a noneconomic policy, i.e. ensuring adequate housing, to have effects on a measure of economic inclusion.

##### Review-level outcome: reducing poverty and economic/material hardship

Three reviews examined the impact of exposures and interventions on reducing poverty and economic/material hardship (Table 3b). One low-quality review [65] and one critically low-quality review [73] identified that unemployment insurance could have positive effects on reducing poverty and material hardship. One of these identified three mechanisms, as follows: that generous eligibility criteria can reduce poverty levels amongst the unemployed, low benefit levels do not reduce poverty as they do not replace wages and flexible eligibility criteria (such as allowing seasonal, migrant or occasional workers) reduces material hardship [73]. The third review (low quality but drawing on high-quality primary studies) identified a positive effect of informal social support for reducing economic/material hardship with low-income mothers in the USA [61]. However, the authors also suggest that the size of the effect was small, and informal support does not benefit other economic outcomes such as employment status, job quality or earnings.

##### Review-level outcome: increased earnings

Only one review examined increased earnings as an outcome (Table 3c). This moderate-quality review (drawing on mostly high- and medium-quality studies) found negligible effects of two types of interventions on the earnings of young people in high-income country contexts, namely skills training (e.g. training in technical/vocational skills, literacy numeracy, soft skills) and employment services (e.g. job search support, mentoring, placements) [60]^6^. There was insufficient data to examine entrepreneurship promotion interventions, and subsidised employment demonstrated a negative effect on earnings. Thus, there is limited synthesised evidence in the review literature to indicate what types of interventions/exposures can increase earnings.

##### Review-level outcome: reducing income inequality

One review examined reduced income inequality as an outcome; however, it was not possible to code an effect direction (Table 3d). This critically low-quality review examined various broad government policies including fiscal policies, education policies, trade liberalisation and labour market reform [74]. However, the review did not provide sufficient detail on the specifics of each type of policy for the effects that they identified to be meaningful. For example, different forms of fiscal policies are discussed (e.g. net expenditure, tax cuts, tax credit reform), but without sufficient detail on the specifics of such policies to be able to evaluate specific effects. Nevertheless, this review met our inclusion criteria and is important as it is one of the few identified which focuses at a macro-level, and it underscores the need for high-quality reviews of specific government policies for their impact on income inequality.

### Equitable access to resources needed to participate in the economy

For the second IE outcome domain, inclusive economy outcomes include access to the resources required to participate in the economy such as early years experiences, health, education and training (Table 1).

#### Types of outcomes and interventions examined by the included reviews

Five reviews examined outcomes that could be categorised as *access to resources to participate in the economy*, but these examined only a limited range of outcomes (Table 2). The most common outcome was improved higher education outcomes (*n* = 3 reviews), one review examined *access* to active labour market programmes and one reviewed the effect of improved entrepreneurial knowledge and skills. No reviews examined other outcomes such as good early years experiences, access to primary or secondary education or vocational training.

For this outcome domain (*access to resources to participate in the economy*), there was no predominant exposure/intervention type, but this domain examined exposures for supporting higher education (such as social/family/peer support and social networks) (*n* = 2 reviews) as well as a mixture of interventions including active labour market programmes (*n* = 2 review), outreach and financial aid interventions (*n* = 1 review). It must be noted that two of these *exposures/interventions* (specifically social networks [75] and active labour market programmes [76, 77] were also examined in the first outcome domain; however, these were by separate reviews, and no single review is included under both sections (i.e. each review only appears in either the ‘Equitable distribution of the benefits of the economy’ or ‘Equitable access to resources needed to participate in the economy’ section). These reviews have been grouped here under this outcome domain as their outcomes are focused on *access to resources*, specifically higher education outcomes [75], *access* to active labour market programmes^7^ and entrepreneurial knowledge and skills.

#### Effect directions identified in the included reviews

We now consider effect directions identified in included reviews in the second outcome domain (Table 4). All the reviews considered here are critically low quality, and the quality of primary studies was not reported.

**Table 4:** Effect directions for exposures/interventions/policies on ‘More equity in access to resources to participate in economy’

*Inclusive economy outcome domain*: More equity in *access to resources needed to participate* in the economy (*n* = 5 reviews)							
	Effect direction			Review characteristics			
Review	Exposure/intervention/policy	Effect direction	Comments	Review quality	PS quality (as reported by review authors)	Total no. of PS with comparator/total PS with IE outcome^a^	Context/population
*3a. Review-level outcome*: Improved higher education outcomes							
** (i)** *Intervention*: Outreach and financial aid interventions for higher education							
[78]	(i) *Outreach interventions* for young people in secondary education such as counselling and tutoring to increase intentions and readiness for transition to higher education	▲	Intervention effective in terms of access as long as more than just information. Less evidence on graduation outcomes	Critically low	Not reported	71/71	Majority of studies (*n* = 59) in North America, (*n* = 6) in Europe, and (*n* = 5) ‘other’ (countries not specified) Disadvantaged students
	(ii) *Financial aid interventions*, i.e. grants, loans, and tax incentives which could be universal or based on need/merit/performance	▲	Impact depends on financial amount and early commitment of aid. Merit-based aid not effective				
	(iii) *Combined interventions*, i.e. a combination of outreach and financial aid interventions	▲					
** (ii)** *Exposure*: Social networks, social capital, and social support							
[75]	*Social networks* (i.e. structure of a set of actors whose members are connected), *social capital* (i.e. the resources linked to having a network) and *social support* (i.e. interactions or relationships which provide attachment/care/love)	▲	Unable to code this ED for specific exposures separately	Critically low	Not reported	Some of 136 but unclear how many	Majority of studies in HIC countries (68% in USA) Underrepresented students
** (iii)** *Exposure*: ‘Enablers’ for transition to higher education							
[72]	‘Enablers’ for HE including the following: Individual abilities, skills and motivations; family & peer support, community and socioeconomic background; social networks, academic integration, extra-curricular activities; educational settings including role of institutions, financial aids, school type	-	Unable to code ED as denominator of PS unclear	Critically low	Not reported	Unclear (191 total PS)	Context unclear although location of authors noted as 49.5% North America, 17.4% Europe, 12.6% South America. Young people in secondary school
*3b. Review-level outcome*: Access to active labour market programmes							
** (i)** *Exposure*: Active labour market programme delivery elements							
[76]	(i) *Training*, i.e. classroom and on-the-job general education or vocational skills	◄►	Mixed findings for low-skilled workers, negative effect on programme access for migrants	Critically low	Not reported	47/47	Germany, Denmark, France, Portugal, Spain, Sweden, Switzerland, Finland, Norway, Poland, the USA, New Zealand, Australia, Austria Low-skilled workers and migrant workers
	(ii) *Job creation programmes*, i.e. the creation of public jobs as part of ‘public works’, e.g. in construction works	◄►	Mixed findings for low-skilled workers, negative effect on programme access for migrants				
	(iii) *Wage subsidies*, i.e. financial incentives given to private employers for hiring those are disadvantaged in the labour market	▼	Negative effects on programme access for both low-skilled and migrants				
*3c. Review-level outcome*: Improved entrepreneurial outcomes, specifically entrepreneurial knowledge and skills							
** (ii)** *Intervention/policy*: Active labour market programmes							
[77]	*Entrepreneurial programmes*, i.e. programmes (including microfinance) intended to support individuals to start their own business	▲	Evidence of effect on increasing knowledge and skills, but RA concludes limited research. Less evaluations of economic outcomes/impact	Critically low	Not reported	1/6	Focused on disadvantaged black male youth in the USA

Key: Effect direction (ED): upward arrow ▲ beneficial impact on IE outcomes, downward arrow ▼ harmful impact on IE outcomes, sideways arrows ◄► no change/mixed effects/conflicting findings/insufficient evidence. *ED* effect direction, *HIC* high-income country, *IE* inclusive economy, *LMIC* low- and middle-income country, *PS* primary study, *RA* review authors, *ROB* risk of bias

^a^Where the number of PS with an IE outcome is not the same as the total number of PS (i.e. non-IE outcomes were considered by some PS) this is noted in brackets

##### Review-level outcome: improved higher education outcomes

Three critically low-quality reviews looked at exposures/interventions to improve higher education outcomes, primarily drawing on studies conducted in North America (Table 4a). The paucity and low quality of reviews in this area and the variety of exposures/interventions examined make synthesising across reviews difficult. Nevertheless, there is some low-quality review-level evidence that some types of outreach and financial aid interventions [78] as well as personal and institutional networks [75] can have positive effects on higher education outcomes for disadvantaged students. However, the specific characteristics of interventions are likely to be important, for example the effectiveness of financial aid depends on whether the financial amount given covers unmet financial need. A range of enablers of the transition to higher education have also been identified (such as the role of family support, socioeconomic background or the role of educational institutions) [72], which could inform intervention development.

##### Review-level outcome: access to active labour market programmes

There was evidence from one critically low-quality review that some disadvantaged groups (low-skilled and migrant workers) could be underrepresented in *accessing* active labour market programmes, but this varied by programme type and group [76] (Table 4b). Although the quality of the primary evidence is unclear, this does suggest the potential for inequalities in accessing programmes intended to support participation in the labour market. Crucially, this was the only review to explicitly focus on *access* to such programmes and to consider *equity* (or lack of) which demonstrates a paucity of evidence in this area. However, this review focused on two specific groups (low-skilled and migrant workers), and a more general consideration of underrepresentation in relation to protected characteristics such as gender, ethnicity or disability was not considered.

##### Review-level outcome: improved entrepreneurial knowledge and skills

Finally, there was some tentative evidence from one critically low-quality review that entrepreneurial programmes could have positive impacts on business knowledge, intending to or actually starting a business, specifically for disadvantaged young Black men in the USA [77] (Table 4c). However, these findings were primarily based on participant self-report rather than measures of economic outcomes, and the authors highlight the very limited evidence base and lack of methodologically robust evaluations.

### Summary of results

Our review of reviews has identified review-level evidence from an inclusive economy perspective, appraised the quality of that evidence and identified key gaps and examined the effects of exposures, interventions and policies on inclusive economy outcomes. The small body of review-level evidence we identified was mostly low quality, and the underlying primary studies were either unknown or mostly low quality. We have also identified a highly heterogenous set of reviews such that (when specific intervention types are considered) there is little overlap between reviews. In addition, significant evidence gaps exist for many inclusive economy outcome domains. That said, we can draw out some key findings.

First, our mapping of review-level outcomes to IE outcome domains showed that the review-level outcomes in the included reviews largely focused on outcomes related to *economic benefits* (*n* = 14) rather than *access to resources* needed to participate in the economy (*n* = 5). Outcomes related to economic benefits were generally limited to a neoclassical economic view of economic benefits [15, 40], such as employment or returning to work, and some limited consideration of measures of income or poverty. Within reviews focused on employment, there was little or no consideration of the quality of work^8^ or stability of income. There was very little focus on economic activity that a more heterodox economic perspective would consider essential to a well-functioning economy, such as the sufficient production and equitable delivery of essential goods and services and participation in the unpaid economy. Those reviews that considered outcomes related to *access to resources needed to participate in the economy* also focused on a limited set of review-level outcomes, primarily access to higher education. Only one review explicitly considered inclusive growth as a concept [74]. Reviews generally focused on specific outcomes, which individually are not sufficient to deliver an inclusive economy.

Second, the identified reviews focused on a limited set of interventions. Across the full set of included reviews, the focus was predominantly on various types of active labour market programmes (*n* = 9) and social security interventions (*n* = 4). Within this, there was some consideration of interventions which might be considered further ‘upstream’, such as focusing on living/working conditions or macro-level policies. Over half of the reviews (*n* = 11) examined interventions focused at the level of the individual, either on interventions intended to improve knowledge and skills.

Third, in terms of intervention effects for the *benefits of the economy*, we identified only three high-quality reviews, and two of these concluded that there was insufficient evidence to draw upon. Of the one high-quality review available, this was still of limited use in terms of our focus on an inclusive economy as it focused on intermediate economic outcomes (i.e. moving into employment), and the review authors acknowledge that the review was more limited in its ability to synthesise evidence on job quality or the longer-term stability of the jobs that people enter. Other reviews did look at outcomes more connected to the fairness of the economy and found that unemployment insurance can have positive effects on reducing poverty and material hardship; however, these reviews were low quality [65, 73]. For active labour market programmes, despite being the most extensively evaluated intervention, much of the review-level evidence is low quality, and issues of low uptake and methodological issues limit our understanding of which specific intervention is most effective in delivering IE outcomes. The higher-quality reviews we identified indicated that there was a lack of good quality evidence to draw upon, whilst some (mostly lower-quality reviews) reported positive intervention effects. Overall, the review-level evidence suggests that programme type is likely to be important.

Fourth, in terms of intervention effects on *access to the resources needed to participate* in the economy, there is much more limited review-level evidence to draw upon (*n* = 5), and this is exclusively drawn from critically low-quality reviews, and there is a lack of overlap in specific intervention types. This means that it is not possible to synthesise across reviews (as we are not comparing ‘like with like’) as intended by the review-of-reviews methodology, and instead, high-quality reviews of primary evidence on specific interventions are indicated.

Finally, the majority of the review-level evidence (*n* = 14) focused on disadvantaged groups, and so the evidence base we have identified is drawn primarily from studies which are not concerned with the general population but with various smaller sub-sections. Furthermore, just two of the included reviews examined review-level outcomes which involved an inequalities/equity dimension: one looked at income inequality [74], and another examined differences in access to active labour market programmes for two minority groups. However, there was a general lack of consideration of the equity impact of exposures, interventions and policies by protected characteristics or by measures of socioeconomic status.

## Discussion

We have identified a small body of review-level evidence (*n* = 19 reviews), of mostly low quality, which examined exposures/interventions/policies for their effects on inclusive economy outcomes. To our knowledge, this is the first review of reviews which examines the review-level evidence base through the lens of an inclusive economy.

The identified reviews focused on a small range of IE outcomes, generally limited to neoclassical view of the economy such as moving people into employment. There was limited focus on action to deliver structural-level reform in the economy; the majority of reviews focused on disadvantaged groups, with much less attention on population-wide interventions. Active labour market programmes and social security interventions were the subject of most reviews. Some positive effects were seen with these programmes on IE outcomes, although programme characteristics were likely to be important, and low uptake limited the impact at the population level.

### How this review fits with existing evidence

Previous overviews have examined ‘upstream’ interventions/policies that impact on population health, including the following: the role of ‘wider’ and macro-economic determinants [16, 79], fiscal policy [20], political factors and political economy (such as the welfare state generosity, political tradition, income inequality [17, 19]), public health policies (including taxing and regulating unhealthy products) [80] and social protection and welfare-to-work policies [81, 82]. However, with one exception [20], existing reviews have focused on health or health inequalities outcomes. Intermediate outcomes (such as employment, poverty, material hardship) are key determinants of health and crucial in addressing health inequalities. Our review examines these key non-health outcomes from the perspective of an inclusive economy.

Our review also identified a general lack of consideration of the *distribution* of outcomes or the equity impact of the identified mechanisms; a crucial finding given equity is a fundamental principle of an inclusive economy [48]. This lack of an equity lens has been noted by others [83, 84].

The included reviews mostly focused on ‘corrective’ interventions for an economic system that has failed particular sub-groups, rather than focusing on altering the economic conditions for the general population to ensure the economy results in greater inclusion and wellbeing ‘by design’ [15, 48].

This resonates with wider health inequalities research, which has demonstrated a tendency for both research and policy to ‘drift downstream’ [85] towards interventions at the level of the individual, and to ‘treat the symptoms rather than the underlying cause of the problem, which may be located in the socioeconomic environment’ [86]. It can be harder to generate empirical evidence for ‘upstream’, macro-level change, particularly as evaluation methods from health research are not appropriate for wider public policy [79, 87]. This has contributed to the ‘inverse evidence law’: ‘we know least about the effects of those interventions that are most likely to influence the wider determinants of health’ [88].

Two recent reviews of reviews have examined the impact of the macroeconomic determinants of health and political economy on population health and health inequalities [16, 17]. A comparatively larger body of review-level evidence was identified, including reviews which examined structural-level factors (such as economic recession, income inequality and the welfare state). In comparison, we have identified a smaller body of review-level evidence. One possible explanation for this difference may be that (systematic) reviews may be a much less well-established methodology in non-health fields.

### Key evidence gaps

There is a lack of reviews examining interventions and policies from the perspective of inclusive growth or inclusive economy. Included reviews did examine outcomes relevant to inclusive economy outcomes, but inclusive economy (or related concepts) was not generally the focus of the reviews. By reducing inclusive economy to its constituent parts, we identified conflicting findings across the reviews. For example, whilst one review identified that unemployment insurance coming to an end can be associated with return to work, two others suggested that unemployment insurance was important for reducing poverty. Complexity and systems thinking in appraising evidence for an inclusive economy might be needed to examine how policies connect and impact on different outcomes in different ways and how different variants of policies may have unintended consequences or negative feedback loops [15, 89, 90].

There is a need for reviews to examine a much wider set of outcomes relevant to an inclusive economy, to move beyond gaining employment as the predominant outcome and consider, for example, quality of work, adequacy and stability of income [91], as well as other economic outputs such as essential goods and services and assets such as wealth [48]. There is also a need for reviews to examine outcomes related to *access to the resources needed to participate* in the economy, such as access to early years/primary/secondary education and training.

The focus on, primarily, ‘corrective’ interventions/policies examined by the reviews suggests that future synthesis should examine a wide range of more ‘upstream’ exposures/interventions/policies in economic development [16, 22]. Fruitful areas for future reviews would include the following: income-based policies such as minimum income standards [92]; good work [2]; community wealth building (including anchor institutions and different forms of ownership models [93]; the delivery of affordable essential services including housing, transport, digital connectivity and food; education, training and skills (e.g. early years, childcare, primary, secondary and further education); social capital and community infrastructure (including the nonpaid economy such as caring, volunteering or mutual aid); community empowerment and engagement; and addressing equitable wealth distribution.

Finally, there is a need for review-level evidence on the equity impact of interventions and policies and how to reduce structural drivers of economic inequalities in characteristics such as gender, ethnicity and socioeconomic status in order that the evidence base considers what types of actions can result in greater economic inclusion for all.

### Strength and weaknesses

This review provides a ‘bird’s-eye view’ of a broad evidence base for researchers and policymakers seeking to advance policies which might support an inclusive economy. However, a number of limitations must be considered.

First, review-level evidence provides only a partial account of the evidence across this field, and further evidence may be available in primary studies which have not yet been included in a review. A review of reviews is reliant on the information reported by review authors [94]. We were not always able to determine whether primary studies had comparators or the quality of primary studies due to incomplete reporting. Many of the included reviews highlighted a lack of primary studies with robust and controlled evaluations, and we were unable to code the effect directions for some interventions due to a lack of studies with a comparator, which limits our findings. Nevertheless, the review-of-review methodology allowed us to assess the scope of a very broad evidence base [52], which would not otherwise have been possible.

Second, it is possible some review-level evidence may be published in databases we did not search. For example, we identified only one review which examined the role of housing^9^ — other evaluations of the impact of affordable housing may be published in specialised town planning or public services literature. Although we set out to examine economic, social and political exposures/interventions/policies, we did not identify any reviews which focused on the relationship between political factors (such as welfare state generosity or political tradition) and inclusive economy outcomes. Again, it may be that our search strategy was not sufficient to capture this type of literature.

Third, we defined a review as one that reported a search strategy in terms of named databases or specified search terms in order that we were able to capture literature from a range of fields and disciplines where review methodology might not apply or may be defined differently. However, this meant that we included some less well-defined reviews, which is likely reflected in the relatively low-quality scores of some of the review papers.

Fourth, the tool we used to assess the quality of included reviews is commonly used in health sciences but may be inappropriate to apply to a field where such tools are not widely applied. Although ideally we would have adjusted the ‘critical weakness’ domains prospectively, these were adjusted post hoc when we considered that we may be underestimating quality by applying stricter criteria. Furthermore, some of our included reviews date back to more than 10 years, prior to (or around the time of) the publication of the first PRISMA statement in 2009, which may explain their lower quality ratings. The degree of awareness of such guidelines in non-health fields is unknown and may also explain the lower quality ratings of some reviews.

Fifth, we are conscious that the amendment to the protocol to exclude reviews focused on low- and middle-income countries (LMIC) may have introduced bias in terms of the gaps identified in the evidence base. There were reviews which covered relevant topics examined in LMIC contexts (such as microfinance) but which were excluded and so are missing from our synthesis of review-level evidence.

Finally, our use of the outcome domains framework [48] is only one way of synthesising the evidence, and we are aware alternative conceptual models may have provided a different perspective. As there no universally agreed conceptual frameworks regarding inclusive economy outcomes or interventions, the gaps we have identified are likely partial and may miss important topics.

### Implications

Three wider points for discussion are important for those working to develop and implement policy actions in this space.

First, there is the perennial issue of the methodological limitations of evaluations of policy actions, particularly as far as ‘upstream’ interventions and policies are concerned. This calls for greater collaboration between public health and economic policy to ensure that research questions and methods align and are fit for purpose in strengthening an evidence base to inform economic policy-making decisions. The necessity for public health to ‘become more economically literate’ [23] and calls for greater collaboration between public health professionals and economists, finance ministries and central banks has been previously articulated [40, 96]. Our review adds to this work and suggests the need for strong, long-term, cross-disciplinary research and policy-focused collaborations.

Second, although we identified a small review-level evidence base, this *does not mean* that there are no primary studies of actions relevant to an inclusive economy. Nevertheless, given that economic policy is being developed, it is necessarily happening in the absence of a clear synthesised evidence base. Systematic reviews of the areas for which there are gaps in the synthesised evidence is clearly needed. In the meantime, it may be worth examining what complimentary types of intelligence may be useful in informing policy decisions and to consider public health’s role in contributing to and appraising this evidence. For example, alongside reviews, scenario modelling of likely policy impacts [97], deliberative democracy approaches [98] and evidence on public values [99, 100] may be important.

Third, outcomes, metrics and measurement are crucial to ensure policies address their intended impacts. However, there is a lack of consensus regarding a clear set of outcomes and metrics for an inclusive economy, to what extent these are informed by citizen perspectives or how best to evaluate trade-offs [101, 102]. To date, review-level evidence has examined a restricted set of outcomes relevant to an inclusive economy, and these largely reflect a neoclassical view of economics. Key measures of an inclusive economy do not appear to be routinely monitored in evaluations of economic policies and interventions, and there is limited consideration of the ‘equity impact’ of such actions. Furthermore, we focused on inclusive economy outcomes, whilst other reviews have considered health outcomes. Combining both health and economic outcomes in future evidence synthesis might help to make trade-offs explicit. A review of ‘welfare-to-work’ policies for lone parents in receipt of social security highlighted negative impacts of such policies on health and wellbeing [81]. This illustrates that the ‘effects’ of interventions are highly dependent on the choice of outcome measure. Those working to develop policy actions to promote an inclusive economy must pay attention to the type of outcome measures chosen to evaluate policies, the need for combining economic and health measures and the equity dimension of evaluations.

## Conclusions

This review has highlighted a small body of mostly low-quality review-level evidence on actions in relation to their impact on inclusive economy outcomes. The current literature focuses largely on ‘corrective’ interventions and policies, primarily focused on supporting disadvantaged groups, rather than ‘upstream’ policies intended to deliver an inclusive economy for the general population ‘by design’. Our review has identified a focus on gaining employment, at the expense of outcomes such as the quality or fairness of work, access to the resources needed to participate in the economy or whether there is greater equity (or not) in such outcomes. This review identifies key gaps in synthesised evidence and highlights the need for greater cross-disciplinary and cross-sectoral collaborations between economics and public health to support evidence-informed economic policy decision-making which can support population health and reduce health inequalities.

### Supplementary Information

**Additional file 1.** Amendments to the protocol.

**Additional file 2.** 2aPRISMA 2020 Checklist. 2bPRISMA 2020 for Abstracts Checklist.

**Additional file 3.** Example Search Strategy (MEDLINE (Ovid)).

**Additional file 4.** Data extraction and Quality assessment templates.

**Additional file 5.** Pilot GRADE assessment for example intervention area: social security.

**Additional file 6.** Summary table of included reviews.

**Additional file 7.** Data extraction and QA for included reviews.

**Additional file 8.** Excluded reviews.

## Data Availability

All data generated or analysed during this study are included in this published article (and its supplementary information files: (1) amendments to the protocol, (2a) PRISMA 2020 checklist, (2b) PRISMA 2020 abstract checklist, (3) example search strategy, (4) data extraction and quality assessment templates, (5) Pilot GRADE assessment, (6) summary table of included reviews, (7) data extraction table for included reviews and (8) table of excluded reviews with reasons for exclusion. The full protocol was published on SocArXiv papers in January 2021 (10.31235/osf.io/dctk5).

## Footnotes

1 ‘Insufficient evidence’ was added to the original categories in the ED template as some review authors had made this explicit.

2 The study design column was removed as all included studies are reviews; ED arrows were not adjusted by sample size, and the study quality key was amended to reflect the AMSTAR-2 categories (high, moderate, low, critically low)

3 Included reviews have an asterisk in the reference list.

4 These totals include some primary studies which were conducted in settings other than high-income countries because of five reviews, whilst the majority of primary studies were conducted in high-income countries, it was not possible to specify how many due to incomplete reporting.

5 The difference between 801 and 525 is largely due to Mocca et al. [72] which reported including 191 primary studies, but it was unclear how many PS focused on an IE outcome as the authors did not provide a list of included studies.

6 Kluve et al. [60] examined both employment outcomes and earnings outcomes and so appears twice in Table 3.

7 In this section, the outcome of interest is *access* to ALMP, and the relevant review [76] looks at which types of ALMP programmes are associated with greater access for marginal groups. In contrast, in the ‘Equitable distribution of the benefits of the economy’ section, the outcomes of interest are employment and earning, and the relevant reviews look at ALMP are a means of achieving this.

8 Kluve et al. [60] considered quality of employment (e.g. contract type, job type) as one of their outcomes of interest; however, there was limited specific consideration of this outcome in the results synthesis; it appears only skills training had clear effects on measures of job equality, and this was more common for low-income country contexts.

9 One notable review on housing improvements was identified but excluded as health was the primary outcome [95].
