# Supplementary material for: Assessing the effects of population-level political, economic and social exposures, interventions and policies on inclusive economy outcomes for health equity in high-income countries: a systematic review of reviews
Source: Syst Rev. 2024 Feb 8;13:58. doi: 10.1186/s13643-023-02429-5 (PMC10851517; doi:10.1186/s13643-023-02429-5)
Supplement: Supplementary file 1 — Additional file 1. Amendments to the protocol. [file 13643_2023_2429_MOESM1_ESM.docx]

**Supplementary File 1: Amendments to the protocol**

A pre-registration form was completed on the Open Science Framework in September 2020 (DOI: [10.17605/OSF.IO/SWT4E)](https://doi.org/10.17605/OSF.IO/SWT4E) and the full protocol published on SocArXiv Papers in January 2021 ([10.31235/osf.io/dctk5)](https://doi.org/10.31235/osf.io/dctk5).

While conducting this review of reviews it was necessary to adapt the full protocol with the following amendments:

**Eligibility criteria:**

1. **Population: Amendment to focus on high-income countries.** In the process of full text screening, it became clear that many potential systematic reviews focused exclusively on low- and middle-income countries (LMIC). Whilst our original protocol stated that *“No restrictions will be applied in terms of geography”,* it was subsequently considered that economies in LMIC would be sufficiently different from high income countries (HIC) that this would complicate the interpretation. Therefore, we made a post-hoc decision to exclude papers which explicitly focused on, or where the majority of papers (i.e., 50% or more) were, LMIC (using the World Bank classification of countries by income) (1). The wording of the Population eligibility criteria were amended to *“High-income countries only. Papers will be excluded which explicitly focused on, or where the majority of papers (i.e., 50% or more) were from low- and middle- income countries (LMIC) using the World Bank classification of countries by income.”*
2. **Intervention(s)-exposure(s): Amendment to ensure consistent focus on ‘exposures, interventions, policies’ rather than ‘mechanisms’ and consistent focus on population-level rather than ‘macro-level’**. Whilst the protocol detailed that the ‘intervention/exposure’ could be *“Any social, political and economic intervention or exposure”*, these were also referred to in the title, aims and objectives as “mechanisms”. For clarity, and because “mechanisms” was difficult to operationalise we have replaced this term with *“exposures, interventions, policies”* which reflects the broad set of factors we are interested in.

In addition, we also ensured that there was consistent reference to *population*-level exposures, interventions, policies, rather than referring to *macro*-level.

1. **Comparator:** In the process of the review, it became clear that the comparison between or within groups was not always evident at the review-level. We therefore updated the eligibility criteria (in Table 1 of the protocol) to specify that reviews must *“include primary studies which”* to make it clear that the comparison was assessed in relation to the study design of the primary studies included by reviews (either as reported by reviews themselves or assessed at the level of primary study). We also identified an editorial error and have removed the following sentence; *“Comparator or control groups are not required for inclusion”* as this contradicted the rest of this criteria.
2. **Outcomes: Amendment to wording of outcomes.** Since the publication of the protocol the outcomes have been refined following further analysis on the attributes on an inclusive economy(2).

The original wording of the outcomes was as follows:

1. opportunities (such as access to education, training, employment, owning and running businesses; owning and managing community assets; finance),
2. the experience of work and the market (e.g. work autonomy, pay, earnings from wealth),
3. the experience of discrimination, and
4. the distribution of the economy’s outcomes (e.g. resources such as quality jobs and living standards, power, income and wealth).

These outcomes have been updated to the following:

Inclusive economy outcomes defined as outcomes that result in more equity in:

1. distribution of the benefits of the economy, specifically (a) essential goods and services, (b) economic inclusion (c) assets that confer economic power and (d) different parts of the economy are valued.
2. access to resources needed to participate in the economy e.g., education, training, employment, etc,.
3. **Study Design:** We have ensured that there is consistent reference to ‘reviews’ rather than ‘systematic reviews’ to reflect our study design requirements (i.e., that an included review must provide some detail on their search strategy around at least search terms used or databases searched) and to ensure that it is clear that our method is a review of reviews.

**Confidence in cumulative evidence**

1. **Amendment not to undertake GRADE:** On the basis of a pilot application, it became clear that with the reviews identified it would not be possible to apply GRADE, and so we decided not to proceed with this aspect of the protocol.

**Data synthesis**

The original protocol stated that findings would be reported narratively and in line with the Synthesis Without Meta-analysis (SWiM) guidelines. As SWiM is typically applied to systematic reviews of primary studies, and as we did not have a set of quantitative effects for each outcome we were not able to follow **all** components of the guidelines. However, we have applied the principles of SWiM as far as possible to increase the transparency of our reporting.

**Aim and objectives, research question and title**

1. **Amendments to wording of aim and objective.** During the review it became clear that it would not be possible to assess “what works” “to deliver” an inclusive economy and therefore the focus of the review had to be amended. This was due to the diverse set of exposures/interventions/policies identified; the inclusion of non-systematic reviews, and because we were unable to undertake GRADE. We have therefore amended the wording of the aim, objectives, and research question to focus on identifying the “effects” of exposures, interventions/policies on inclusive economy outcomes.

The original objectives were to:

- *“contribute to the evidence base on what works for delivering an inclusive economy with regard to populations and context i.e. what works for whom and in what context, and*
- *identify the quality of existing evidence and areas where evidence is missing to inform future work in this area.”*

These have been amended to:

- *“to synthesise review-level (synthesised) evidence of the effects of exposures/interventions/policies on inclusive economy outcomes*
- *Assess the quality of existing reviews and areas where reviews are missing to inform future work in this area.”*

1. **Amendment to wording of research question.** In line with point 7 above we amended the wording of the research question. The original question was:

*“What are the population-level political, economic and social mechanisms that can be used to deliver an inclusive economy?”*

This was changed to:

*“What are the effects of population-level political, economic and social exposures, interventions and policies on inclusive economy outcomes?”*

1. **Title: Based on the changes in points 1, 2, 7 and 8 above we have amended the wording of the title.**

This was changed from:

*“Assessing the effects of macro-level political, economic and social exposures, interventions and policies on inclusive economy outcomes in high income countries: A protocol for a systematic review of reviews.”*

To the following wording:

*“Assessing the effects of population-level political, economic and social exposures, interventions and policies on inclusive economy outcomes in high income countries: A protocol for a systematic review of reviews.”*

1. The World Bank. The World by Income and Region  [Internet]. The World by Income and Region. [cited 2022 Apr 20]. Available from: https://datatopics.worldbank.org/world-development-indicators/the-world-by-income-and-region.html#:~:text=The%20World%20by%20Income%20and%20Region%20The%20world,income%20groups%3A%20low%2C%20lower-middle%2C%20upper-middle%2C%20and%20high%20income.?msclkid=a96c2e26c0b711eca8e6bb55b237eb4d

2. Shipton D, Sarica S, Craig N, McCartney G, Katikireddi SV, Roy G, et al. Knowing the goal: an inclusive economy that can address the public health challenges of our time. J Epidemiol Community Health. 2021 Nov;75(11):1129–1132.
