# Supplementary material for: Assessing the effects of population-level political, economic and social exposures, interventions and policies on inclusive economy outcomes for health equity in high-income countries: a systematic review of reviews
Source: Syst Rev. 2024 Feb 8;13:58. doi: 10.1186/s13643-023-02429-5 (PMC10851517; doi:10.1186/s13643-023-02429-5)
Supplement: Supplementary file 3 — Additional file 3. Example Search Strategy (MEDLINE (Ovid)). [file 13643_2023_2429_MOESM3_ESM.docx]

**Supplementary File 3: Example Search Strategy (MEDLINE (Ovid))**

Searches from 1946 to 26 May 2020

| [# ▲](http://ovidsp.dc1.ovid.com.knowledge.idm.oclc.org/sp-4.05.0b/ovidweb.cgi?&S=EILFFPMKNGACBDBPKPBKDGHOBKCCAA00&Sort+Sets=descending) | **Searches** | **Results** |
| --- | --- | --- |
| 1 | (capital or demand?side or supply?side or fiscal polic* or fiscal responsibilit* or tax* or redistributive polic* or redistributive effect* or distributive polic* or trade polic* or monetary polic* or interest rate or inflation or deflation or money suppl* or credit suppl* or financial polic* or deficit or national budget or national deficit or national debt or social policy or econom* or macroeconom* or economic integration or recession or austerity or capitalis* or socialis* or neoliberal* or Marx* or Keynes* or financial economy or microeconomic polic* or rent?seeking or lobby* or voting behavio?r or fiscal polic* or political econom*).ab. or (capital or demand?side or supply?side or fiscal polic* or fiscal responsibilit* or tax* or redistributive polic* or redistributive effect* or distributive polic* or trade polic* or monetary polic* or interest rate or inflation or deflation or money suppl* or credit suppl* or financial polic* or deficit or national budget or national deficit or national debt or social policy or econom* or macroeconom* or economic integration or recession or austerity or capitalis* or socialis* or neoliberal* or Marx* or Keynes* or financial economy or microeconomic polic* or rent?seeking or lobby* or voting behavio?r or fiscal polic* or political econom*).ti. | 598317 |
| 2 | (decentral* or de-central* or local authorit* or local government* or local council* or municipalit* or public infrastructure investment or social polic* or social investment state or (nationali?ation or privati?ation) or size of government or public ownership or industrial polic* or industriali?ation or industrial structure or regulation or deregulation or government polic* or public goods or public fund* or welfare program* or infrastructure or procurement or local tax* or public polic* or government or labo?r polic*).ab. or (decentral* or de-central* or local authorit* or local government* or local council* or municipalit* or public infrastructure investment or social polic* or social investment state or (nationali?ation or privati?ation) or size of government or public ownership or industrial polic* or industriali?ation or industrial structure or regulation or deregulation or government polic* or public goods or public fund* or welfare program* or infrastructure or procurement or local tax* or public polic* or government or labo?r polic*).ti. | 1000118 |
| 3 | (labo?r market or labo?r market reform* or market power or market design or free?market or oligopol* or market regulation* or monopoly or market structure* or financial market* or externalit* or employment or unemployment or wage* or informal econom* or underground econom* or employment or unemployment or labo?r econom* or labo?r market discrimination or value of life or labo?r supply or labo?r demand).ab. or (labo?r market or labo?r market reform* or market power or market design or free?market or oligopol* or market regulation* or monopoly or market structure* or financial market* or externalit* or employment or unemployment or wage* or informal econom* or underground econom* or employment or unemployment or labo?r econom* or labo?r market discrimination or value of life or labo?r supply or labo?r demand).ti. | 75613 |
| 4 | (welfare state or benefits or social security or social safety net or social protection or social floor or social welfare or welfare or household welfare or welfare program*).ab. or (welfare state or benefits or social security or social safety net or social protection or social floor or social welfare or welfare or household welfare or welfare program*).ti. | 349129 |
| 5 | (bargaining power or collective bargaining or unioni?ation or trade union* or economic participation or social participation or political participation or workplace participation or labo?r managed firm* or employee ownership or producer cooperative*).ab. or (bargaining power or collective bargaining or unioni?ation or trade union* or economic participation or social participation or political participation or workplace participation or labo?r managed firm* or employee ownership or producer cooperative*).ti. | 4890 |
| 6 | (private sector or public sector or third sector or NGO* or non?government* organi?ation* or non?profit organi?ation* or voluntary sector or civic sector or non?profit sector or community sector or public enterprise* or public?private enterprise* or private enterprise* or formal sector or informal sector or shadow econom* or informal institution*).ab. or (private sector or public sector or third sector or NGO* or non?government* organi?ation* or non?profit organi?ation* or voluntary sector or civic sector or non?profit sector or community sector or public enterprise* or public?private enterprise* or private enterprise* or formal sector or informal sector or shadow econom* or informal institution*).ti. | 20979 |
| 7 | (firm* or bank* or depository institution* or business cycle or business fluctuation* or externalit* or business growth or co?operative* or firm behavio?r or behavior of firm* or organi?ation behavio?r or behavior?r of organi?ation* or firm choice or transaction cost* or business tax* or subsid* or capitalist enterprise* or co?operative enterprise*).ab. or (firm* or bank* or depository institution* or business cycle or business fluctuation* or externalit* or business growth or co?operative* or firm behavio?r or behavior of firm* or organi?ation behavio?r or behavior?r of organi?ation* or firm choice or transaction cost* or business tax* or subsid* or capitalist enterprise* or co?operative enterprise*).ti. | 189606 |
| 8 | (credit union* or debt* or financial inclusion or financial exclusion or financial stability or income or mortgage or microfinance or lending or financial service* or household saving* or personal finance* or household behavio?r or consumer economics or income or wealth or capital or intangible capital).ab. or (credit union* or debt* or financial inclusion or financial exclusion or financial stability or income or mortgage or microfinance or lending or financial service* or household saving* or personal finance* or household behavio?r or consumer economics or income or wealth or capital or intangible capital).ti. | 166949 |
| 9 | (digital or technolog* or innovation or social innovation or intellectual capital or intellectual property or open data or intellectual commons or information commons).ab. or (digital or technolog* or innovation or social innovation or intellectual capital or intellectual property or open data or intellectual commons or information commons).ti. | 596660 |
| 10 | (entrepreneur* or human capital or skill* or education or training or social capital or social contract or food security or personal income).ab. or (entrepreneur* or human capital or skill* or education or training or social capital or social contract or food security or personal income).ti. | 916409 |
| 11 | (natural capital or de?growth or gross domestic product or GDP or gross national income or gross value added or GVA or earth capitalism or environmental econom* or (productivity or production) or land reform or housing or property right* or discriminat* or intersectionality or vulnerab* or exploit* or public commons or commons).ab. or (natural capital or de?growth or gross domestic product or GDP or gross national income or gross value added or GVA or earth capitalism or environmental econom* or (productivity or production) or land reform or housing or property right* or discriminat* or intersectionality or vulnerab* or exploit* or public commons or commons).ti. | 1478033 |
| 12 | (economic democracy or economic equality or solidarity economy or fair economy or just economy or economic inclusion or inclusive econom* or inclusive growth or pro?poor growth or shared prosperity or shared growth or equity or justice or equality or inequalit* or poverty or poverty reduction or reduc* poverty or economic inequalit* or income inequalit* or power inequalit* or wealth inequalit* or income equalit* or power equalit* or wealth equalit* or distribution of power or distribution of wealth or distribution of income or resilient econom* or economic resilience or wellbeing econom* or well-being econom* or resilient cit* or urban resilience or resilient development or inclusive institution* or economic impact or impact of econom* or increas* access to opportunities or increas* participation in the economy or economic participation or equitable distribution benefits or social justice or good work or fair work or fair income or job security or financial inclusion or reduc* discrimination or reduc* vulnerability or address* intersectionality or reduc* intersectionality or reduc* oppression or reduc* exploitat* or access to education or access to training or plural ownership or distributed ownership or distributed control or democratic control or asset equality or asset inequalit* or distribution of asset* or fair distribution of benefits or income growth).ab. or (economic democracy or economic equality or solidarity economy or fair economy or just economy or economic inclusion or inclusive econom* or inclusive growth or pro?poor growth or shared prosperity or shared growth or equity or justice or equality or inequalit* or poverty or poverty reduction or reduc* poverty or economic inequalit* or income inequalit* or power inequalit* or wealth inequalit* or income equalit* or power equalit* or wealth equalit* or distribution of power or distribution of wealth or distribution of income or resilient econom* or economic resilience or wellbeing econom* or well-being econom* or resilient cit* or urban resilience or resilient development or inclusive institution* or economic impact or impact of econom* or increas* access to opportunities or increas* participation in the economy or economic participation or equitable distribution benefits or social justice or good work or fair work or fair income or job security or financial inclusion or reduc* discrimination or reduc* vulnerability or address* intersectionality or reduc* intersectionality or reduc* oppression or reduc* exploitat* or access to education or access to training or plural ownership or distributed ownership or distributed control or democratic control or asset equality or asset inequalit* or distribution of asset* or fair distribution of benefits or income growth).ti. | 101151 |
| 13 | ((systematic review or scoping review or meta?analys* or overview or literature review or review of literature or critical review or review of recent literature or synthesis of evidence or analytical review or research synthesis or meta?regression or realist review) not book review).tw. | 451564 |
| 14 | 1 or 2 or 3 or 4 or 5 or 6 or 7 or 8 or 9 or 10 or 11 | 4597641 |
| 15 | 12 and 13 and 14 | 2916 |
| 16 | limit 15 to (english language and humans) | 2021 |
