# Supplementary material for: Assessing the effects of population-level political, economic and social exposures, interventions and policies on inclusive economy outcomes for health equity in high-income countries: a systematic review of reviews
Source: Syst Rev. 2024 Feb 8;13:58. doi: 10.1186/s13643-023-02429-5 (PMC10851517; doi:10.1186/s13643-023-02429-5)
Supplement: Supplementary file 4 — Additional file 4. Data extraction and Quality assessment templates. [file 13643_2023_2429_MOESM4_ESM.docx]

**Supplementary File 4: Data extraction[[1]](#footnote-1) and Quality assessment templates**

**(i) Data extraction template**

**Information about the review**

**Review ID**

Enter Cochrane study ID along with Primary author last name and publication year. If author name not available, enter lead organisation/research group name.

**Review publication date**

Please enter the publication date (year) for the review. Note that this will usually be in the Review ID.

**Review title**

Title of paper / abstract / report that data are extracted from

**Lead author contact details**

Please note any/all of the following: phone number, email address or full address.

Please type 'Not provided' if not provided.

**Publication status**

1. Peer-reviewed
2. Pre-print
3. Other grey literature, e.g. NGO and governmental reports

**Review funder**

Please note 'Not provided' if not provided in the publication.

**Possible conflict of interest as reported by review authors.**

Please type in 'None' if authors declared no conflicts of interest. Type in 'Not provided' if no declaration was available in the publication.

**Any other note(s)**

Fill this note with notes on synthesis or anything else that is not covered by this table.

**Review methods**

**Original review aim(s)**

Please type in, or paste from the original publication, the aim(s) of the original review as reported by the review authors. If the review forms a part of a larger piece of work, please note the aim for the review section only.

**PICO(S) elements**

**Population**

What was the population of interest of the review?

If the authors did not make a statement and this cannot be inferred from the whole paper, please note 'Not provided/Unclear'. If there are no restrictions, please note 'No restrictions'.

Please note that as our review is looking at population-level factors, most identified reviews are likely to focus on countries instead of specific populations. In such cases, please note the country/region as interest.

**Original Intervention/Exposure**

What was the specific intervention(s)/exposure(s) of interest of the main review?

If the authors did not make a statement and this cannot be inferred from the whole paper, please note 'Not provided/Unclear'. If there are no restrictions, please note 'No restrictions'.

**Interventions/Exposure relevant to inclusive economy outcome only**

It may be possible that some reviews looked at a subset of interventions/exposures for different outcomes. Here, please note the specific intervention/exposure of interest in relation to the inclusive economy outcomes.

If the authors did not make a statement and this cannot be inferred from the whole paper, please note 'Not provided/Unclear'. If there are no restrictions, please note 'No restrictions'. If this is the same as the intervention/exposures listed for the main review, please note 'Same as the main review'.

**Definition and measures of Intervention/Exposure relevant to inclusive economy outcomes**

This question looks for two answers: (1) Definition of the intervention/exposure(s), and (2) Measure(s) for the intervention(s)/exposure(s).

For each, if not applicable, i.e. the review reported no interventions/exposures of interest, please type in 'not applicable'.

If authors do not provide specific definition(s)/measure(s) for the intervention(s)/exposure(s), please type in 'not provided'.

Sample answer: (1) Definition: not provided, (2) Measure: income inequality as measured by Gini coefficient.

**Comparison/Comparator**

Did the primary papers included in the review need to have a comparison/comparator for inclusion? Comparisons could be within or between groups.

If the authors did not make a statement and this cannot be inferred from the whole paper, please note 'Not provided/Unclear'. If there are no restrictions, please note 'No restrictions'.

**Original outcomes of interest in the review**

In this section, please list all original outcomes of interest in the review. This may include non-inclusive economy outcomes.

If the authors did not make a statement and this cannot be inferred from the whole paper, please note 'Not provided/Unclear'.

**Inclusive economy outcome(s)**

Please specify the outcome of interest of the review. If authors cover multiple inclusive economy outcomes, please include all of these. Please extract information only on inclusive economy outcomes in this section.

If the authors did not make a statement and this cannot be inferred from the whole paper, please note 'Not provided'. If there are no restrictions, please note 'No restrictions'. If this is the same as the outcomes listed for the main review, please note 'Same as the main review'.

**Inclusive economy outcome(s) definition**

This question looks for two answers: (1) Definition of inclusive economy outcome(s), and (2) Measure(s) for inclusive economy outcome(s)..

For each, if not applicable, i.e. the review reported no interventions/exposures of interest, please type in 'not applicable'.

If authors do not provide specific definition(s)/measure(s), please type in 'not provided'.

Sample answer: (1) Definition: not provided, (2) Measure: income inequality as measured by Gini coefficient.

**Primary study design**

E.g. RCT, non-randomised observational study, etc.

If the authors did not make a statement and this cannot be inferred from the whole paper, please note 'Not provided/Unclear'.

**Time period between intervention/exposure and outcome of interest**

Please report if the review specified a time period that must have elapsed between the intervention/exposure and the outcome, i.e. were they interested in longitudinal studies? Cross-sectional ones? This is similar to the comparison question but focuses more on time than group comparisons.

Please note 'Unclear' if no details were reported. In some cases, comparison may be inferred from the paper.

Sample answer, e.g. Unclear. Likely to be no restrictions based on the methods and discussion section.

**Time period of interest at review-level**

Was the review restricted to searching studies covering a specific time period?

**Publication period**

Was the review interested in finding studies published in a specific time period? This may be different than the 'time period of interest at review-level'. e.g. inception of each database until the date of last search

Type in 'Unclear' if this was not reported in the review.

**Search methods**

**Language restriction(s)**

**Number of databases searched**

Please type in 'Unclear' if not clear from the publication.

**Name of databases searched**

Please list all databases searched. Type in 'Unclear' if the complete list is not clear.

**Date of the last searches**

What was the date of last search?

**Other methods**

**Underlying theory/framework**

Did the authors provide A PRIORI theory/framework for the relationship between the interventions(s)/exposure(s) and inclusive economy outcome of interest? Please note that this is different to the mechanism question under the 'Review-level findings' section. This question focuses on a priori theories.

Enter 'none' if none was provided.

**Synthesis methods**

1. Quantitative: e.g. Meta-analysis
2. Narrative synthesis
3. Realist synthesis
4. Other

**Review-level findings**

**Number of primary studies included in the review**

Enter the total number of primary studies the review included.

**Number of primary studies focusing on an IE outcome**

Enter the number of primary studies focusing on at least one IE outcome.

Type in 'same as the main review' if the number focusing on IE outcomes was the same as the total number of studies included in the review.

**Date of earliest primary study focusing on IE outcome**

Please type in the publication year of the earliest primary study focusing on IE outcome. Report date(s) separately for each outcome where the review focuses on multiple outcomes.

**Date of latest primary study focusing on IE outcome**

Please type in the publication year of the latest primary study focusing on IE outcome. Report date(s) separately for each outcome where the review focuses on multiple outcomes.

**Main results**

Please summarise the main findings of the review.

Please extract results only for inclusive economy outcomes of interest.

**Quantitative results**

Please extract the exact data on quantitative effect measures/summary measures, if applicable. This is likely to be more relevant for studies undertaking meta-analysis than those doing narrative synthesis.

Please type in 'Not applicable' if there were no quantitative effect measures reported.

**Context for the main results**

**Country/setting**

Please note the country/setting of the primary studies from which each finding was derived.

Please detail the context for each inclusive economy outcome and/or exposure/intervention separately. Context may include details of the following as reported in the main review: geography, democracy type, specific population group, specific time period.

Sample answers. (1) role of X policy on Y: positive in LMIC while no effect in other countries

(2)Role of W on Z: positive in the 90s, no effect in the 2000s.

(3) A not associated with B in X subgroup.

Note that this field is different to the overall setting of the review as it is specific to each finding. Please type in 'Not applicable' if there is nothing relevant to report for this field.

**Time period**

Where, applicable please note the specific time period primary studies covered for a particular finding. This is different to the review-level time period.

Please type in 'Not applicable' if there is nothing relevant to report for this field.

Sample answers: X tended to improve Y during 1990s, but no similar effect was observed in the 2000s.

**Other (e.g. methodology related context)**

Please detail any other caveats that need to be considered when interpreting the results. Please type in 'Not applicable' if there is nothing relevant to report for this field.

Sample answer: Observed effects of X on Y depended on the statistical method adopted in each primary paper.

**Making sense of results**

**Proposed mechanisms**

Detail if a review proposed mechanisms between the intervention/exposure and inclusive economy outcome of interest. Please extract data separately for each inclusive economy outcome of interest. If a review focuses on multiple exposures or mechanisms, please extract data separately for each. Please type 'Not reported' if no mechanisms are proposed in the review.

**Consistency of findings**

At a review-level, were there any inconsistencies in findings? If yes, did the authors comment on reason for these?

Sample answer 1: Specific intervention tended to have different outcomes in different settings. Authors have explicitly commented on this and provided xxx as an explanation.

Sample answer 2: Review found mixed results of the impact of exposure X on Y. Authors did not attempt to recognise or find reasons for these inconsistencies.

**Primary study data quality**

Did the review comment on/assess primary study data quality? If available, provide details on overall primary study data quality. If further details are available, provide primary study quality per inclusive economy outcome of interest.

Please extract data on primary studies relevant to inclusive economy outcomes only. If disaggregated quality not available per outcome of interest, comment on overall quality of primary studies included in the review, where possible.

If the review does not attempt to assess quality of primary studies, please note 'Not reported'.

**Evidence gaps identified in the review**

Were there any gaps in evidence identified in the review? If yes, please detail them. Type in 'None' if authors didn't find any evidence gaps.

Please extract data only on inclusive economy outcomes of interest.

**(ii) Quality assessment template**

**Did the research questions and inclusion criteria for the review include the components of PICO?**

PDF version of the form here: https://www.bmj.com/content/bmj/suppl/2017/09/21/bmj.j4008.DC1/sheb036104.wf1.pdf

Detailed guidance when judging each domain here (PLEASE READ BEFORE STARTING ASSESSMENT): https://www.bmj.com/content/bmj/suppl/2017/09/21/bmj.j4008.DC1/sheb036104.ww1.pdf

1. Yes
2. No

*Extractors will also be able to add supporting text to justify their judgements*

***Did the report of the review contain an explicit statement that the review methods were established prior to the conduct of the review and did the report justify any significant deviations from the protocol?**

CRITICAL POINT.

Please note that the guidance asks you to find the published protocol and compare it against the published review report if the answer is yes.

1. Yes
2. Partial yes
3. No

*Extractors will also be able to add supporting text to justify their judgements*

**Did the review authors explain their selection of the study designs for inclusion in the review?**

1. Yes
2. No

*Extractors will also be able to add supporting text to justify their judgements*

***Did the review authors use a comprehensive literature search strategy?**

CRITICAL POINT.

1. Yes
2. Partial yes
3. No

*Extractors will also be able to add supporting text to justify their judgements*

**Did the review authors perform study selection in duplicate?**

1. Yes
2. No

*Extractors will also be able to add supporting text to justify their judgements*

**Did the review authors perform data extraction in duplicate?**

1. Yes
2. No

*Extractors will also be able to add supporting text to justify their judgements*

***Did the review authors provide a list of excluded studies and justify the exclusions?**

CRITICAL POINT Note: this was changed from a ‘critical weakness’ to ‘weakness’

1. Yes
2. Partial yes
3. No

*Extractors will also be able to add supporting text to justify their judgements*

**Did the review authors describe the included studies in adequate detail?**

1. Yes
2. Partial yes
3. No

*Extractors will also be able to add supporting text to justify their judgements*

***Did the review authors use a satisfactory technique for assessing the risk of bias (RoB) in individual studies that were included in the review?**

CRITICAL POINT.

Unless the primary studies included in the review were randomised controlled trials, please follow instructions for NRSI when judging this domain.

1. Yes
2. Partial yes
3. No

*Extractors will also be able to add supporting text to justify their judgements*

**Did the review authors report on the sources of funding for the studies included in the review?**

1. Yes
2. No

*Extractors will also be able to add supporting text to justify their judgements*

***If meta-analysis was performed did the review authors use appropriate methods for statistical combination of results?**

CRITICAL POINT. Note: as most reviews did not conduct a meta-analysis this was not considered a critical weakness.

This is unlikely to be relevant in the reviews included in out umbrella review. However, it is a critical point where relevant.

1. Yes
2. No
3. No meta-analysis conducted

*Extractors will also be able to add supporting text to justify their judgements*

**If meta-analysis was performed, did the review authors assess the potential impact of RoB in individual studies on the results of the meta-analysis or other evidence synthesis?**

Unless the primary studies included in the review were randomised controlled trials, please follow instructions for NRSI when judging this domain.

1. Yes
2. No
3. No meta-analysis conducted

*Extractors will also be able to add supporting text to justify their judgements*

***Did the review authors account for RoB in individual studies when interpreting/discussing the results of the review?**

CRITICAL POINT

1. Yes
2. No

*Extractors will also be able to add supporting text to justify their judgements*

**Did the review authors provide a satisfactory explanation for, and discussion of, any heterogeneity observed in the results of the review?**

1. Yes
2. No

*Extractors will also be able to add supporting text to justify their judgements*

***If they performed quantitative synthesis did the review authors carry out an adequate investigation of publication bias (small study bias) and discuss its likely impact on the results of the review?**

CRITICAL POINT Note: as most reviews did not conduct a meta-analysis this was not considered a critical weakness.

1. Yes
2. No
3. No meta-analysis conducted

*Extractors will also be able to add supporting text to justify their judgements*

**Did the review authors report any potential sources of conflict of interest, including any funding they received for conducting the review?**

1. Yes
2. No

*Extractors will also be able to add supporting text to justify their judgements*

**Rating of overall confidence in the review (critical weaknesses are highlighted with an asterisk in the above questions)**

High - Zero or one non-critical weakness: The systematic review provides an accurate and comprehensive summary of the results of the available studies that address the question of interest

Moderate - More than one non-critical weakness: The systematic review has more than one weakness, but no critical flaws. It may provide an accurate summary of the results of the available studies that were included in the review.

Low - One critical flaw with or without non-critical weaknesses: The review has a critical flaw and may not provide an accurate and comprehensive summary of the available studies that address the question of interest.

Critically low - More than one critical flaw with or without non-critical weaknesses: The review has more than one critical flaw and should not be relied on to provide an accurate and comprehensive summary of the available studies.

*Note: Multiple non-critical weaknesses may diminish confidence in the review and it may be appropriate to move the overall appraisal down from moderate to low confidence

1. High
2. Moderate
3. Low
4. Critically low

*Extractors will also be able to add supporting text to justify their judgements*

1. The data extraction form was piloted with five included reviews to resolve any misunderstandings and to finalise the data extraction form. As a result the data extraction fields were not changed, but additional instructions added to a couple of questions [↑](#footnote-ref-1)
