# Supplementary material for: Assessing the effects of population-level political, economic and social exposures, interventions and policies on inclusive economy outcomes for health equity in high-income countries: a systematic review of reviews
Source: Syst Rev. 2024 Feb 8;13:58. doi: 10.1186/s13643-023-02429-5 (PMC10851517; doi:10.1186/s13643-023-02429-5)
Supplement: Supplementary file 5 — Additional file 5. Pilot GRADE assessment for example intervention area: social security. [file 13643_2023_2429_MOESM5_ESM.docx]

**Supplementary File 5: Pilot GRADE assessment for example intervention area: social security**

The protocol stated that an adapted version of the GRADE approach would be undertaken to assess the confidence in the cumulative evidence base (Guyatt et al., 2011). We tested the application of the method set out in the protocol for one intervention area, namely social security. Whilst it was possible to provide an initial ranking of the theme based on quality assessment of included reviews, step 2 required assessment across seven domains, and for five of these domains there was insufficient evidence to inform an assessment of upgrading or downgrading. A key issue was that the theme considered three different outcomes, and two different types of exposure/intervention, and so there was not one ‘effect’ under examination. Furthermore, as only one review undertook meta-analysis, we lacked the quantitative data necessary to assess precision, magnitude etc. Based on this pilot application, we decided not to proceed with an assessment of the confidence in cumulative evidence.

**Step 1**: Initial ranking based on overall quality of included reviews (one high quality (Filges et al., 2013), two low- quality (Barr et al., 2010; Renahy et al., 2018) and one low-quality (O’Campo et al., 2015). Overall, this theme was ranked as *“low quality: Further research is very likely to change conclusions or sufficient body of evidence does not exist to understand the true effect”*

**Step 2:** Revisit initial ranking and upgrade/downgrade according to several domains.

Table 1: Adapted GRADE domains (from original protocol)

| **Domain** | **Actions** | **Assessment for ‘income’ theme** |
| --- | --- | --- |
| Quality of included reviews and primary studies in the reviews (as reported in the reviews) | The lower the quality of the reviews and primary studies, the lower the confidence in cumulative evidence. Consider downgrading initial rating if quality is low. | One SR reported low quality primary studies (Barr et al., 2010). One SR was realist review and did not assess study quality (O’Campo et al., 2015). One SR excluded low quality studies (Filges et al., 2013). One SR reported 3 primary studies assessed as weak, moderate and strong (Renahy et al., 2018). Insufficient evidence to justify ‘upgrading’. |
| Inconsistency of impact | The higher the inconsistency of the impact of the intervention/exposure in the same context, the lower the confidence in cumulative evidence. Consider downgrading initial rating if inconsistency is apparent. | The included reviews consider three different outcomes – employment, reducing poverty, and reducing economic / material hardship and evaluate two different exposures – disability benefits (Filges et al., 2013; Barr et al 2010) and unemployment insurance (O’Campo., 2015; Renahy et al., 2018). Therefore, not one intervention/exposure and not one outcome so difficult to assess consistency of effect across the theme. |
| Imprecision | The higher the quantitative precision around the effect of the intervention, the higher the confidence in cumulative evidence. | Only one review undertook meta-analysis (Filges et al., 2013) so unable to assess precision of the effect across the theme. |
| Publication/reporting bias | The higher the risk of within-review and inter-review publication bias, the lower the confidence in cumulative evidence. Consider downgrading initial rating if it is likely that statistically non-significant findings remain unpublished | Only one review (Filges et al., 2013) assessed publication bias so unable to assess publication bias risk across the theme. |
| Magnitude of effect | Consider upgrading initial rating is observed effect is particularly large. | Only one review undertook meta-analysis (Filges et al., 2013) so unable to assess magnitude of effect across the theme. |
| Dose-response gradient | Upgrade if evidence indicates an apparent dose-response gradient. | Only one review undertook meta-analysis (Filges et al., 2013) so unable to assess dose-response gradient across the theme. |
| Confounding | Consider whether there are unmeasured confounders in the body of evidence and whether these confounders are likely to only reduce the observed effect. If yes, consider upgrading initial rating. | Most of the primary studies included in the SRs are observational. Possibility of uncontrolled confounders raised by Barr et al., (2010). Insufficient evidence to justify upgrading initial rating. |
