# Supplementary material for: Assessing the effects of population-level political, economic and social exposures, interventions and policies on inclusive economy outcomes for health equity in high-income countries: a systematic review of reviews
Source: Syst Rev. 2024 Feb 8;13:58. doi: 10.1186/s13643-023-02429-5 (PMC10851517; doi:10.1186/s13643-023-02429-5)
Supplement: Supplementary file 6 — Additional file 6. Summary table of included reviews. [file 13643_2023_2429_MOESM6_ESM.docx]

**Supplementary File 6: Summary table of included reviews (in alphabetical order)**

| **Review**  **(Author & date)** | **Population and**  **Country/setting** | **Intervention/exposure** | **Inclusive economy outcomes** | **Main results relevant to IE outcomes** |
| --- | --- | --- | --- | --- |
| **Bambra et al (2005)** | Individuals of working age (16-59/64 years) with a moderate physical or mental illness/disability    United Kingdom | Welfare-to-work interventions in the UK, specifically five different interventions:  1) education, training and work placements;  2) vocational advice and support services;  3) in-work benefits for employees;  4) employer incentives, and  5) improving accessibility of work environment. | Moving into paid employment. | The review concluded that the welfare-to-work programmes evaluated supported people with disabilities into work but that as only a minority of the primary studies were controlled it is difficult to know if this was due to programme effectiveness or wider factors. |
| **Barr et al (2010)** | Working age adults (16-69 years) with health problems or disabilities.    Canada, Norway, Sweden, United Kingdom | Changes to generosity / eligibility requirements of disability benefits. | Probability of being in employment    Length of time off work or not in employment | Only 1 of 5 relevant studies suggested that changes in eligibility had an impact on employment (p.1111)  8 of 11 studies suggested increased generosity reduces labour market participation, but the authors highlight lack of high-quality evidence to assess degree of effect. |
| **Bassuk et al (2014)** | Homeless families.    United States | Housing interventions and service interventions | Employment | Some improvements in employment status, but most were not receiving a living wage. |
| **Clayton et al (2011)** | Working age adults (16-65 years) with limiting long term illness or disability who were not employed and were on some form of incapacity-related benefit.    United Kingdom | National, major ‘Welfare-to-work’ and ‘return to work’ programmes – focused on the individual | Employment chances and social inclusion | Personal advisors and case management could help participants back to work but these results were affected by selection bias of more *“work-ready claimants”* (p.8).  Despite being national programmes the take-up of programmes was low. Financial incentives were set too low. |
| **Clayton et al (2012)** | Working age adults (16-65 years) who are chronically ill or disabled.    Canada, Denmark, Norway, Sweden and the UK | ‘Major’ / national ‘Welfare-to-work’ and ‘long-term sick to work’ programmes – focused on the employer. | Employment chances and social inclusion | The most promising interventions were financial incentives for employers, workplace adjustments and programmes to require employers to engage in return-to-work planning.  However, these initiatives may not impact at the population level due to low uptake and awareness. |
| **Bonoli & Liechti (2018)** | Low-skilled workers and migrant workers    Germany, Denmark, France, Portugal, Spain, Sweden Switzerland, Finland, Norway, Poland, USA, New Zealand, Australia, Austria | Active labour market policies focused on three types of programme: Training; job creation; wage subsidies | *Access* to labour market programmes | Varying levels of underrepresentation/access depending on programme type and target group as well as the type of welfare regime.  Training – both groups experienced were underrepresented but more underrepresentation for migrants.  Job creation programmes – low skilled workers had greater access, but migrant workers were underrepresented.  Wage subsidies – underrepresentation for both groups.  Social democratic welfare states showed more positive access compared to conservative welfare states. |
| **Filges et al (2013)** | Unemployed individuals who had received unemployment benefit with a known exhaustion date.    European countries, United States and Canada | Exhaustion of any kind of unemployment benefit with a known expiration date | Gaining employment. | Evidence that approaching ending of unemployment benefit increased moving into employment i.e., an exhaustion effect in the month/week of benefit exhaustion. |
| **Gensby et al (2012)** | Employees on sick leave due to physical injury, illness or mental health disorders.    North America | Workplace based disability management programmes | Primary outcomes: return to work; sickness absence duration; reduction in days absent from work. | Lack of evidence to draw firm conclusions regarding effectiveness of programs due to lack of effect sizes for primary studies and high risk of bias for the two non-randomised studies. |
| **Hayday et al (2008)** | All adults (16+ years) living in the community who are unemployed because of long-term incapacity^[[1]](#footnote-1)^ and/or in receipt of incapacity benefit/disability benefits or other similar benefit.    United Kingdom | Interventions, programmes, policies to help recipients of incapacity benefit return to work | Return to work | A lack of UK-based evidence which evaluates the effectiveness of interventions to support people receiving incapacity/disability benefits to return to work. |
| **Herbaut & Geven (2020)** | Disadvantaged students  Majority of studies (n=59) in North America, (n=6) in Europe and (n=5) ‘Other’ (countries not specified). | Outreach interventions and  Financial aid interventions | Enrolment or completion of higher education (HE) | Outreach interventions – effects are mixed depending on type of programme.  Information and support - can have positive impact on access to HE but information only or intensive academic tutoring less effective.  Financial aid interventions – effects are mixed depending on the type of aid.  Needs-based grants improve graduation rates but less positive impact on enrolment rates. Merit based grants less effective.  Combined interventions – positive effects on both enrolment and graduation. |
| **Heshmati et al (2019)** | Not provided | Any governmental policy initiative aiming to tackle income inequality | Income inequality | Four key factors were identified that have been used by governments to address income inequality: fiscal policy; education policy; trade liberalization (although its negative effects are recognised); and labour market reform. The review covers diverse types of these policies and results are not synthesised in terms of impact of specific policies on income inequalities. |
| **Jennings (2014)** | Economically disadvantaged African-American male youths (15-24 years)    United States | Entrepreneurial and microenterprise development initiatives | Entrepreneurial and economic impact outcomes | Entrepreneurial training was associated with higher scores for knowledge, qualitative reports of start-ups or scores for entrepreneurial activities (based on the two studies with a comparator). However, the authors highlight a lack of robust evaluations of entrepreneurial / microenterprise interventions for this population. |
| **Kluve et al (2017)** | Young women and men (15 to 35 years)    58% from high-income countries; 42% from low-income countries | Active labour market programmes of four types:  1) training and skills development;  2) entrepreneurship promotion (including access to credit/microfinance);  3) employment services;  4) subsidized employment. | Employment and earnings | Overall positive effects on employment and earnings but the effects are small and there is variation between programmes.  The effects were generally smaller in high-income countries compared to low-income countries. |
| **Mishra et al (2020)** | Higher education students with a focus on under-represented students    United States (68%), Canada, Netherlands, UK, Australia, Spain, Italy, Norway, Russia and ‘other’ countries with one study each. | Social network, social capital and social support | Higher education success | The review suggested better social networks have positive relationship with higher education success. |
| **Mocca et al (2019)** | Young people in secondary school, university/vocational institutes, and the labour market | Any enabler and or barrier to transition to Higher Education | Transition to Higher Education | Key enablers identified:  Individual abilities - personal ambition; Microsystem - family support, peer support, socioeconomic background);  Mesosystem - academic integration, social networks, extra-curricular activities) and Exosystem - academic support, facilitative role of institutions; financial aids, school type). |
| **O’Campo et al (2015)** | Working age adults.    OECD countries  (Particularly Central and Eastern European Countries, Canada and the United States) | Unemployment insurance | Short- and longer-term poverty and material hardship | Three important factors identified:  Generous eligibility criteria – poverty in the unemployed is reduced.  Low benefit levels – poverty not reduced.  Flexible eligibility criteria– material hardship is reduced. |
| **Ott & Montgomery (2015)** | Individuals who meet the domestic legislative definition of a refugee for the country of the intervention | Interventions intended to increase economic self-sufficiency and wellbeing of refugees | Primary outcome: Employment rate or labour force participation rate. | No results as no included studies – empty review. |
| **Radey et al (2018)** | Low-income mothers    United States | Social support / informal support | Economic / material hardship | Informal support associated with less economic hardship, material hardship and need for public assistance, but the size of the impact of informal support likely to be small. |
| **Renahy et al (2018)** | Working population in high income countries    North America and Nordic European countries | Unemployment insurance | Economic difficulties in the previous year; relative poverty and absolute poverty | Although differences between studies, overall, evidence for a beneficial effect of unemployment insurance on poverty. |

1. Note the term ‘incapacity’ used by the review authors; however we recognise that this review was published some time ago, and this may not be the most acceptable term in use now. [↑](#footnote-ref-1)
